# Entropy of Branching Out: Linear versus Branched Alkylthiols Ligands on CdSe Nanocrystals

**DOI:** 10.1021/acsnano.1c10430

**Published:** 2022-02-14

**Authors:** Orian Elimelech, Omer Aviv, Meirav Oded, Xiaogang Peng, Daniel Harries, Uri Banin

**Affiliations:** † The Institute of Chemistry and The Center for Nanoscience and Nanotechnology, The Hebrew University of Jerusalem, Jerusalem 9190401, Israel; ‡ Department of Chemistry, Zhejiang University, Hangzhou 310027 P. R. China; § The Fritz Haber Center, The Hebrew University of Jerusalem, Jerusalem 9190401, Israel

**Keywords:** CdSe nanocrystals, ligand exchange, branched ligands, isothermal titration calorimetry, conformational entropy

## Abstract

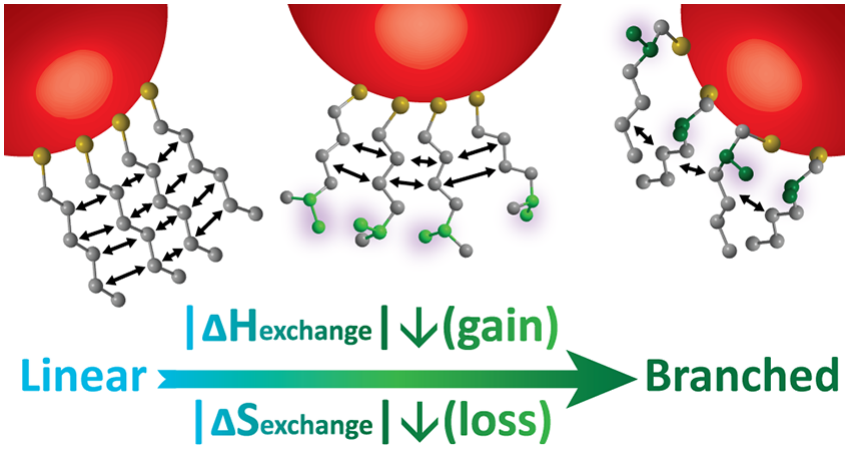

Surface ligands of semiconductor nanocrystals (NCs) play key roles in determining their colloidal stability and physicochemical properties and are thus enablers also for the NCs flexible manipulation toward numerous applications. Attention is usually paid to the ligand binding group, while the impact of the ligand chain backbone structure is less discussed. Using isothermal titration calorimetry (ITC), we studied the effect of structural changes in the ligand chain on the thermodynamics of the exchange reaction for oleate coated CdSe NCs, comparing linear and branched alkylthiols. The investigated alkylthiol ligands differed in their backbone length, branching position, and branching group length. Compared to linear ligands, lower exothermicity and entropy loss were observed for an exchange with branched ligands, due to steric hindrance in ligand packing, thereby justifying their previous classification as “entropic ligands”. Mean-field calculations for ligand binding demonstrate the contribution to the overall entropy originating from ligand conformational entropy, which is diminished upon binding mainly by packing of NC-bound ligands. Model calculations and the experimental ITC data both point to an interplay between the branching position and the backbone length in determining the entropic nature of the branched ligand. Our findings suggest that the most entropic ligand should be a short, branched ligand with short branching group located toward the middle of the ligand chain. The insights provided by this work also contribute to a future smarter NC surface design, which is an essential tool for their implementation in diverse applications.

Surface ligands have long been recognized for their many roles and functionalities dictating the characteristics of semiconductor nanocrystals (NCs).^1,2^ Beyond their use in controlling the NC size and shape,^3−6^ they prevent agglomeration,^7−12^ provide electronic passivation of the NC surface,^13−17^ and are used to tune the solubility of NCs in various media (polar/nonpolar),^18−22^ serving as the basis for the broad applicability of semiconductor NCs enabled *via* bottom-up chemical manipulation.^23−27^ Therefore, a myriad of studies were aimed at studying various properties of the NCs surface ligand layer.^28−30^

A useful method for studying the role of the ligand is by using NCs surface ligand exchange reactions, as they allow for surface manipulation in a relatively simple approach. Nuclear magnetic resonance (NMR) analysis,^31−39^ as well as fluorescence measurements,^40−42^ provided extensive knowledge regarding the exchange mechanism and also some indirect information about the reaction thermodynamics. Recently, isothermal titration calorimetry (ITC) was established as a powerful tool for studying the thermodynamics of surface reactions in NCs as a direct measuring technique.^43−51^ ITC allows for following the heat changes associated with a step-by-step ligand exchange reaction, thus allowing for the extraction of a complete set of thermodynamic parameters.

In a previous work, we studied the ligand exchange reaction of oleate coated CdSe NCs with a homologous series of linear alkylthiols.^47^ The heat detection sensitivity of the ITC (0.1 μJ) enabled the comparison between ligand exchange reaction of alkylthiols, varying in chain length with an even number of carbon atoms in the backbone. A higher enthalpy gain was observed for the longer alkyl chain, indicating an increase in the interchain van der Waals interactions between bound ligands,^52,53^ which serves as a driving force for their denser packing,^54^ relative to the native oleate packing. On the contrary, this tight packing resulted in a higher entropy loss with increasing ligand length, revealing a compensation mechanism between the enthalpy and entropy. Similar conclusions were reported by Calvin *et al*. for the reaction between oleate coated InP NCs and a set of either linear phosphonic acids, or linear carboxylates, monitored trough ITC.^50^ The phosphonic binding group was also studied by Gee *et al*.,^48^ with a ligand exchange reaction between oleate coated CdSe NCs and alkylphosphonic acids. In their study, a sequential route involving a charge ligand exchange reaction followed by a neutral ligand binding reaction was observed.

While studying the effect of the binding group is important for the NCs electronic properties,^55−58^ it has been already established that the ligand tail is essential for not only the NC synthesis and its colloidal stability^59−61^ but also determining the thermodynamic route in surface modifications.^47,50^ However, most of the studies so far have varied in their binding group, with moderate changes in the ligand tail, mostly relating to the length of the backbone but not addressing the possible effects of structural changes in the ligand tail.^62^

Recently, Peng and co-workers reported that branched-chain surface ligands enhance NCs solubility by a few orders of magnitude compared to those capped with conventional linear surface ligands.^60^ Branching of the alkyl chain is assumed to interrupt ligand interdigitation between adjacent NCs and maximizes the intramolecular entropy of the solvated NCs, making the dissolution of aggregates thermodynamically favorable, thus enhancing the colloidal stability.

Herein, we investigate the differences between branched ligands and linear ligands, mostly focusing on the changes in the overall entropy of the system between them. We monitored on-surface ligand exchange reactions between oleate (−O_2_CR′) coated CdSe NCs and a series of linear versus branched alkylthiol ligands (RSH):
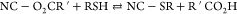
1

While both ligand series included backbone lengths ranging from butanethiol (BT) to decanethiol (DT), the branched ligand series also included ligands with varying branching positions along the backbone chain, as well as methyl and ethyl branching groups, as will be elaborated later on. Since the commercial availability of branched alkylthiols is limited, most of the studied ligands were synthesized in our lab (see Section 3 of the Supporting Information (SI) for additional information). The thermodynamic parameters of the exchange reaction were extracted following ITC experiments (Figure 1). Our studies revealed that, upon ligand exchange reaction, the branched ligand systems demonstrated reduced exothermicity and lower loss of entropy, in comparison to the exchange with linear ligands. This supports the initial assumption that NCs coated with branched ligands should demonstrate increased stability.

**Figure 1:**
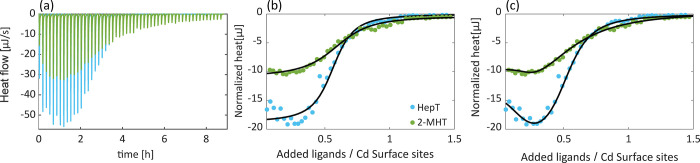
ITC results for ligand exchange reaction of oleate coated CdSe NCs (*d* = 3.0 nm) with 2-methyl-1hexanethiol (2-MHT, green) and the corresponding linear chain with similar total number of carbons 1-heptanethiol (HepT, blue) at 323 K. (a) Real-time ITC thermogram. (b and c) Titration curve of the heat change as a function of the mole fraction of added ligand to Cd^2+^ surface sites (green circles). The data were fitted to a “ligand exchange” model with (b) one or (c) two types of independent binding sites (black lines). See text for additional details.

Special focus was given to the contribution of the conformational entropy to the overall entropy change upon ligand binding. By using mean-field theory previously applied to lipid bilayers as well as micellar systems,^63−66^ we were able to calculate the entropy of dispersed chains in solution and on the NC, where conformations are subject to ligand packing constraints. Despite the similarity of the ligand layer to micellar systems, little has been done toward applying those well-established theoretical tools to analyze phenomena at the NC interface. Thus, in our work, we applied and augmented this theoretical framework to our system and, by that, were able to formulate predictions that go beyond the scope of our experimentally investigated branched ligands. This has allowed us to resolve the origin of the conformational entropy increase attributed to those ligands. Specifically, our calculations indicate that ligands with branching groups located toward the middle of the chain should demonstrate higher intramolecular entropy when bound to the NC surface. This should, in turn, affect the NCs colloidal stability, since ligand shell order/disorder was reported as a crucial parameter for preventing NC agglomeration.^7−12^

## Results and Discussion

Figure 1a presents a typical ITC thermogram, measured for the exchange reaction of oleate coated CdSe NC (*d* = 3 nm) with 2-methyl-1-hexanethiol (2-MHT, green) and with the corresponding linear ligand, 1-heptanethiol (HepT, blue). The HepT molecule, which has a similar total number of carbons to 2-MHT, was chosen as a comparable system to allow for the isolation of the branching effect from the effects related to the number of carbons. The heat flow represents an exothermic reaction, as expected for the exchange of a carboxylate ligand with an alkylthiol. Integration of the thermogram peaks resulted in a titration curve, representing the heat change as a function of the ratio between the added ligand and the Cd^2+^ surface sites (Figure 1b). The curves of both ligands are fitted to an independent single site exchange reaction model, recently presented by us.^47^ This model considers both the detachment of the native oleate ligand and the binding of the alkylthiol and allows for the extraction of a single set of thermodynamic parameters including the enthalpy change (Δ*H*), the entropy change (Δ*S*), and Gibbs free energy change (Δ*G*). Similar to our previous work, for all investigated ligands, the exchange reaction is spontaneous (Δ*G* < 0) and involves heat release (Δ*H* < 0) and entropy loss (Δ*S* < 0). A more detailed explanation regarding this model can be found in the SI (Section 5).

The exchange with the branched ligand is less exothermic (Δ*H* = −13.0 kJ/mol) and involves lower loss of entropy (Δ*S* = −14.9 J/molK), relative to the exchange with the corresponding linear ligand (Δ*H* = −22.4 kJ/mol and Δ*S* = −37.6 J/molK). Within the ligand layer surrounding the NCs surface, the branched methyl group of the 2-MHT ligand induces steric hindrance, which reduces the formation of van der Waals interactions between neighboring ligands, in comparison with the nonbranched linear ligands (HepT). Hence, a decrease in the reaction exothermicity is observed. The lower entropy loss upon exchange with branched ligands compared with the corresponding linear ligands may also be attributed to the steric hindrance in packing, which grants a higher degree of motion for the bound ligands.

While the single-site model reasonably represents the branched 2-MHT titration curve, the curve of the linear ligand (HepT) exhibits some deviations from this model, suggesting an existence of an additional type of site. Figure 1c presents the fitting of both curves with an independent two-site ligand exchange model, which was recently presented by us. As was interpreted in our previous work, the two sites are associated with the facet sites and the edge sites on the NCs surface.^47,67,68^ According to the model, the sites in minority are characterized by lower enthalpy gain (−16 and −13 kJ/mol for HepT and 2-MHT, respectively) and lower entropy loss (−4 and −3 J/molK for HepT and 2-MHT, respectively) along with a more negative Δ*G* (−14 and −11 kJ/mol for HepT and 2-MHT, respectively). This is expected for edge sites, which do not allow for dense packing of the ligands and hence result in smaller van der Waals interaction manifested by the lower enthalpy gain, while also providing a higher degree of motion expressed by the lower loss of entropy. In addition, the higher accessibility of those sites for exchange induces a higher binding affinity, which therefore resulted in a more negative Δ*G*. The sites in majority, which are correlated with the facet surface sites, are typified by a higher exothermicity (−30 and −21 kJ/mol for HepT and 2-MHT, respectively) and higher entropy loss (−53 and −68 J/molK for HepT and 2-MHT, respectively) due to the denser packing of the ligands, leading to elevated van der Waals interactions and lower degrees of freedom upon ligand exchange. Moreover, these sites are less available and hence exhibit lower binding affinity, thus resulted in less negative *ΔG* (−8 and −4 kJ/mol for HepT and 2-MHT, respectively). When comparing both models and their fits (Tables S5 and S6), it is noticeable that the single-site model represents an average behavior of two sites, which is not always manifested in a good fit, while the two-site model not only provides a more accurate fitting to the collected data but also allows for deeper understanding of the changes in the ligand arrangement on the different sites of the NCs surface. Hence, despite the fact that for some curves the interpretation of the results under the single-site model is sufficient, for consistency, all the analysis presented here on will be interpreted using the two-site model.

## Effect of Branching Position

**Figure 2:**
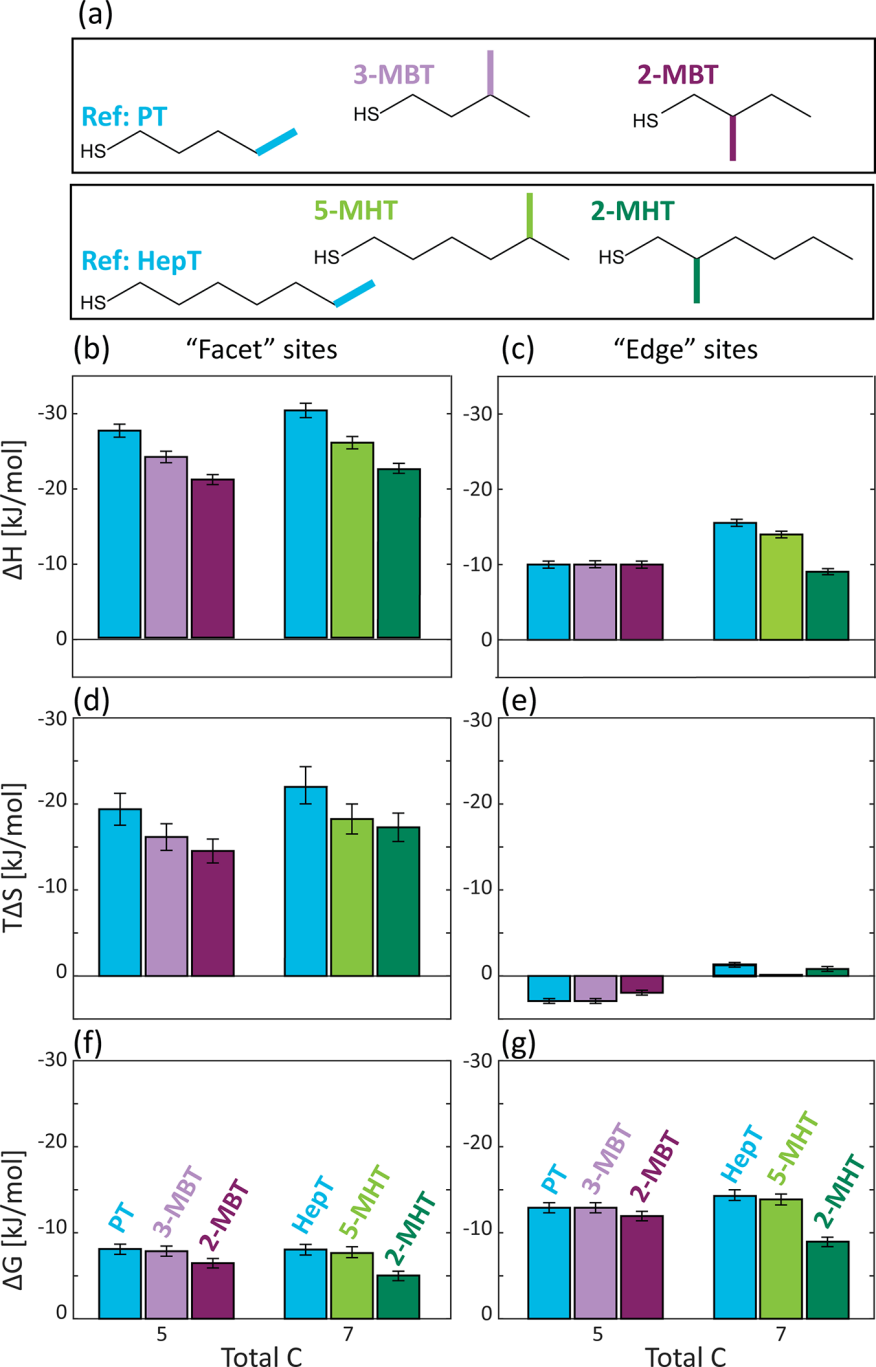
Effect of methyl branching position on the thermodynamics of the ligand exchange reaction. (a) Structures of the investigated branched ligands with total number of carbons of 5 (light purple, 3-MBT; dark purple, 2-MBT) and 7 (light green, 5-MHT; dark green, 2-MHT). (b–g) Thermodynamics parameters extracted upon fitting the ITC curves with a two-site exchange model: (b and c) enthalpy, (d and e) entropy, and (f and g) Gibbs free energy changes for facet (b, d, and f) and edge (c, e, and g) surface sites, respectively. Results are compared to the corresponding linear alkylthiols, with a similar total number of carbons (blue).

To study the effect of the branching position of the methyl group (Figure 1, green), two groups of ligands were chosen: (I) two branched butanethiols, 3-methyl-1-butanethiol and 2-methyl-1-butanethio (3-MBT and 2-MBT, respectively, Figure 2a, purple), with 1-pentanethiol (PT) for reference as a linear (*i*.*e*., non branched) ligand with the same total number of carbons; (II) two branched hexanethiols, 5-methyl-1-hexanethiol and 2-methyl-1-hexanethiol (5-MHT and 2-MHT, respectively, Figure 2a, green), with linear 1-heptanethiol (HepT) as a reference. The thermodynamic parameters, Δ*H*, *T*Δ*S*, and Δ*G* for each ligand exchange reaction, extracted by fitting the ITC data to a two-site exchange model, are presented in Figure 2b–g in a column bar representation mode. Errors of the extracted parameters were determined by the quality of the data fitting to the exchange model, combined with the reproducibility of the measurements (see Section 5.3.1 of the SI).

Looking into the parameters associated with the facet sites (*i*.*e*., main sites, Figure 2b,d,f), we notice several prominent trends. With regards to the enthalpy of the exchange reactions (Figure 2b), the highest enthalpy gain was measured for the reaction with the linear ligand (blue columns). This is expected since the formation of van der Waals interactions between neighboring branched ligands is interfered by the presence of the methyl branching group, in contrast to the interactions between adjacent linear ligands. The exchange with ligands that possess a branching methyl group near the surface anchoring group, such as 2-MBT and 2-MHT, was found to be the least exothermic within their set (Figure 2b, dark purple and dark green columns, respectively). This suggests that the formation of van der Waals interactions is more hindered in the presence of a methyl branching group located in close proximity to the NC surface. This explanation also complies with the observations recorded for the entropy change upon ligand exchange (Figure 2d). The highest loss of entropy is detected for the linear ligands, while the smallest is detected for the branched chains with branching group near the surface. Whereas the packing of linear molecules upon binding to the NCs surface causes a major loss in the system’s degrees of freedom, this loss is less pronounced for the branched ligands, due to the methyl branching group, leading to a sparser packing. An increase in the packing disorder originating from branching in different positions was already reported for phospholipid bilayers by Poger *et al*.^69^ In this work, the introduction of a methyl group along the phospholipid chain led to unfavorable steric interactions between the additional methyl and the rest of the hydrocarbon chain, locally disrupting the packing of the membrane. This disruption was maximal when the branching group was located toward the middle of the chain and negligible for iso-branched chains, as the terminal methyl groups are intrinsically disordered. Similarly, the changes observed for the thermodynamic parameters of the iso-alkylthiol ligands in our work, 3-MBT and 5-MHT (Figure 2 dark purple and dark green columns correspondingly), are mostly subtler than those of 2-MBT and 2-MHT, respectively. The lower exothermicity and the slightly smaller entropy loss measured for 2-MBT and 2-MHT (non-iso ligand, Figure 2 light purple and light green columns correspondingly) suggest a loose packing and formation of weak van der Waals interactions within these ligand layers.

Comparing the Gibbs free energy of the exchange reaction between the linear and the branched ligands, we observed a decrease in the absolute value of Δ*G* for the non-iso branched alkylthiols (*i*.*e*., 2-MBT and 2-MHT, Figure 2f). We can probably associate the moderated free energy to the steric hindrance arising from the branching methyl group, preventing ligand penetration and arrangements on/to the ligand layer and therefore decreasing the ligand affinity to the NC surface.

The observed changes in thermodynamic parameters due to the addition of a branching methyl group are significant, with up to 25% drop in the entropy loss compared with that observed for the linear ligands (Figure 2d). The ITC results presented here confirm the ability of branched ligands to indeed harvest high entropy, even upon binding to the NC surface, further substantiating their labeling as “entropic ligands”.

Generally, the changes in the thermodynamic parameters of the edge sites are moderate, yet the observed trends are similar to the facet sites. This is expected since the ligand packing on the NC edges is less dense and the intramolecular interactions are weaker to begin with. (Figure 2c,e,g). These observations were common to all the ligands measured; thus, from here on, we will focus our discussion on trends observed for the main facet site.

## Effect of Branching Group Length

**Figure 3:**
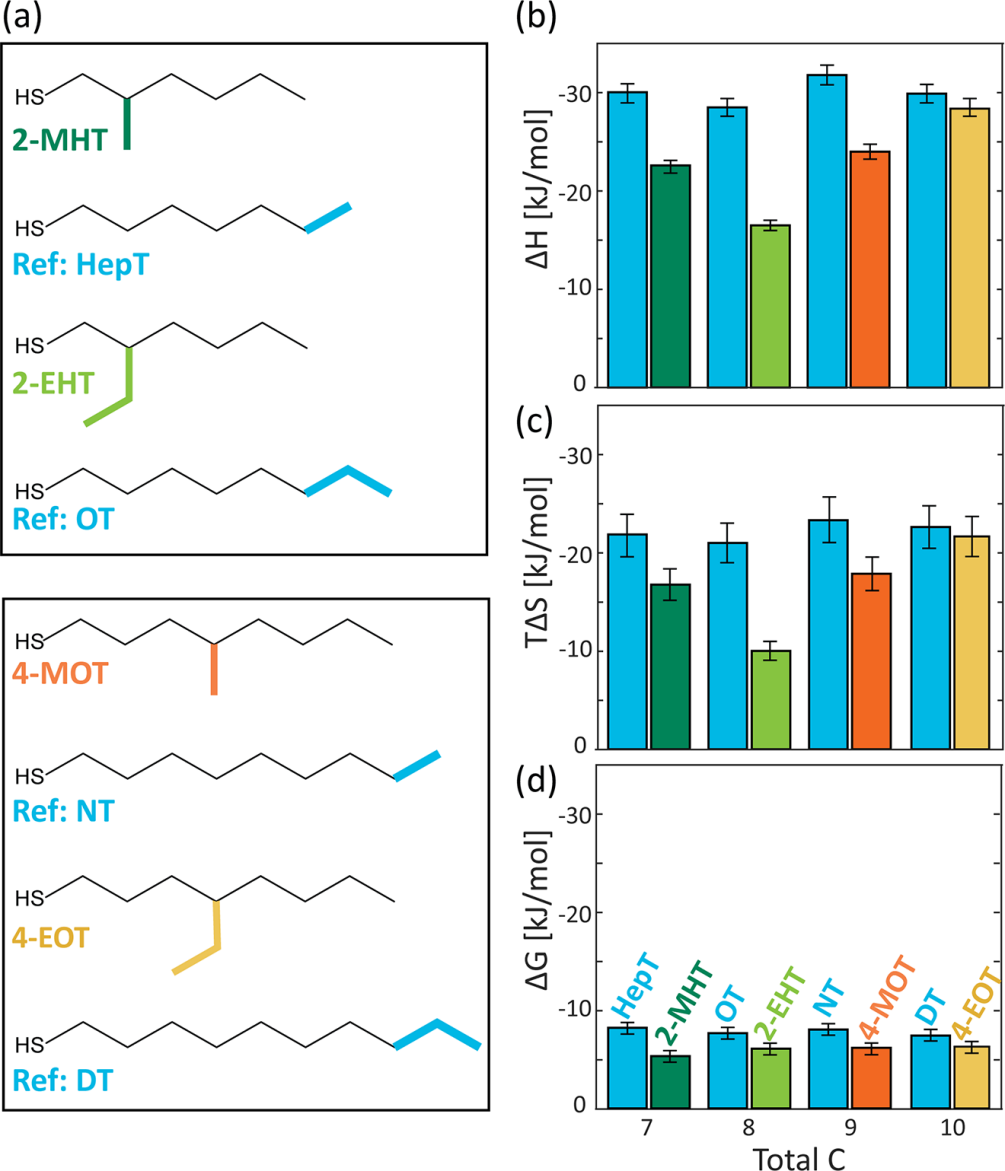
Effect of branching group length on the thermodynamics of the ligand exchange reaction. (a) Structures of investigated branched ligands with backbone lengths of 6 (green) and 8 (orange) carbons. (b) Enthalpy, (c) entropy, and (d) Gibbs free energy changes extracted for facet surface sites upon fitting the ITC curves with a two-site exchange model. Results are compared to the exchange with corresponding linear alkylthiols, with similar total number of carbons (blue).

In order to determine the effect of the branching group length, we chose two sets of ligands where in each set the branching position and the backbone length were kept identical and only the branching group was altered to be either -methyl or -ethyl. The first chosen set comprises branched ligands with a hexanethiol backbone: 2-methyl-1-hexanethiol (2-MHT, compared with heptanethiol, HepT; Figure 3a, dark green and blue, respectively) and 2-ethyl-1-hexanethiol (2-EHT, compared with octanethiol, OT; Figure 3a, light green and blue, respectively). The second set includes branched ligands with an octanethiol backbone: 4-methyl-1-octanethiol (4-MOT, compared with nonanethiol, NT; Figure 3a, orange and blue, respectively) and 4-ethyl-1-octanethiol (4-EOT, compared with decanethiol, DT; Figure 3a, yellow and blue, respectively).

The extracted thermodynamics parameters for the facet surface sites are presented in Figure 3 (for the edge site parameters see Tables S5 and S6). Generally, all branched ligands present the “branching effect” of a decrease in the exothermicity and in the entropy loss, compared with the corresponding linear ligands. As for the effect of varying the branching group in each set, opposite trends were observed for each backbone set. While the exothermicity and entropy loss of the 2-EHT are significantly lower than the parameters measured for the 2-MHT, an opposite trend is observed between 4-MOT and 4-EOT. This is attributed to the interplay between the steric hindrance for the ligand backbone packing, induced by the branching group and the packing possibility of the branched group itself. As observed earlier, when the branching group is located near the binding group (the case of 2-MHT and 2-EHT), there is a strong disruption for ligand packing arising from the branching group. This disruption even increases for the 2-EHT ligand, resulting in a decrease in both the exothermicity and in the entropy loss. Moreover, for the 2-EHT ligand, indication of two different sites in the titration curve is washed out (Figure S9). This is probably due to the high steric hindrance, which minimizes the difference in ligand packing between facet and the edge sites, causing a uniform packing. Most likely, the ligand organization in the case of 2-EHT is analogous to the packing on the edges, according to the thermodynamics parameters (Table S6). The changes in Gibbs free energy support this assumption, as its value is slightly more negative for 2-EHT than 2-MHT. This may appear counter to our previous conclusion that branched ligand exhibits a lower affinity to the NC surface (lower absolute value of Δ*G*) due to the steric hindrance for penetration. However, as 2-EHT tends to form an “edge-like” packing, it is less hindered and therefore results in a slightly higher binding affinity and thus more negative Δ*G*.

For the 4-MOT and 4-EOT system, in which the branching position is located farther away from the NC surface, the 4-EOT ligands have a possibility to form van der Waals interactions with the ethyl branching group as well (either with neighboring ligand backbones or even with neighboring ethyl branching groups). Hence, an increase in both the enthalpy gain and the entropy loss is observed. The difference in Gibbs free energy between 4-MOT and 4-EOT is minute and within the error of the fitting.

## Effect of Chain Backbone Length

**Figure 4:**
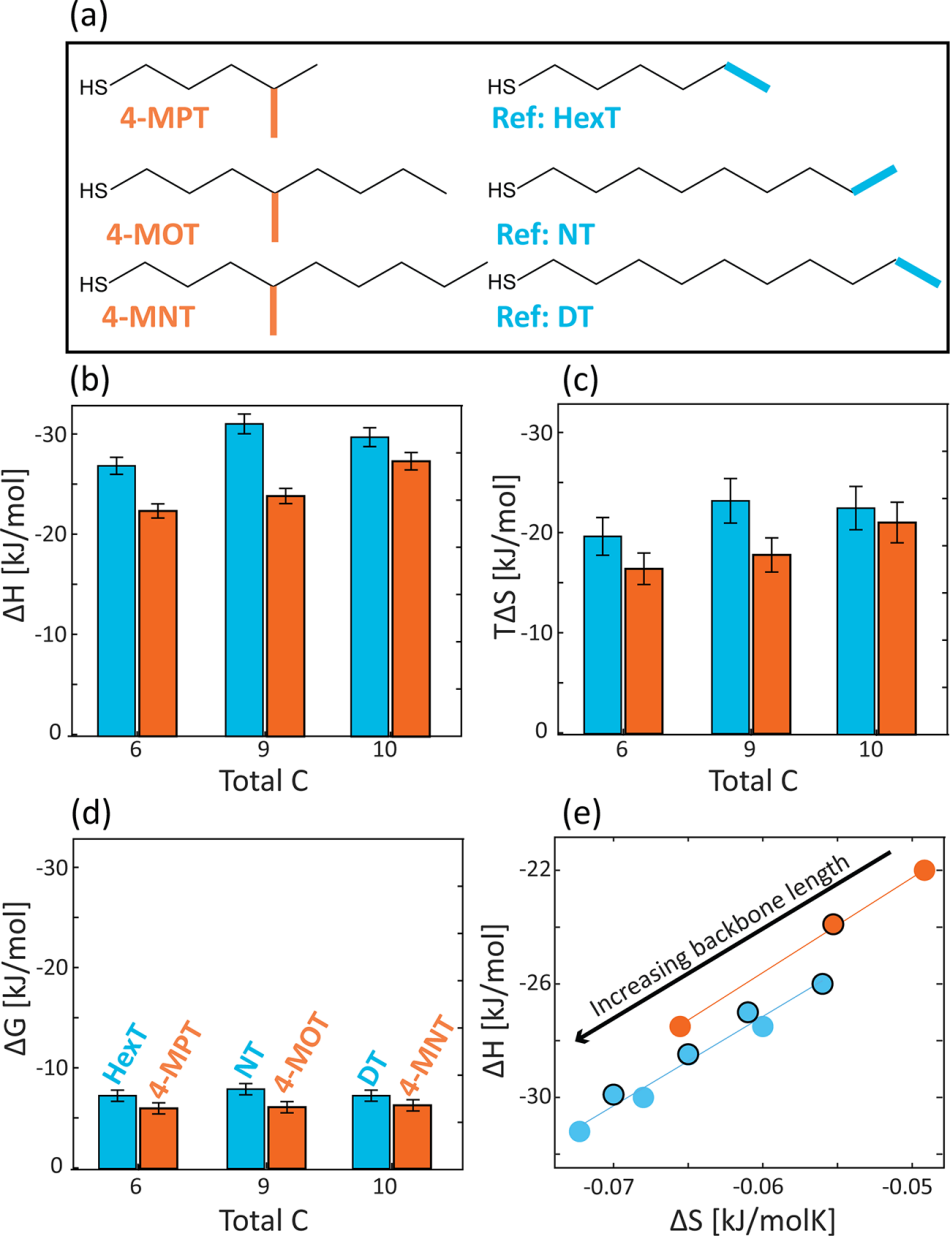
Effect of backbone length on the thermodynamics of the ligand exchange reaction with 4-methyl branched ligands. (a) Structures of investigated branched ligands with varying backbone length (orange). (b–d) Thermodynamic parameters extracted upon fitting the ITC curves with a two-site exchange model: (b) enthalpy, (c) entropy, and (d) Gibbs free energy changes for facet surface sites. Results are compared to the exchange with corresponding linear alkylthiols, with a similar total number of carbons (blue). (e) Enthalpy–entropy compensation plotted for linear (blue, chain length of 4–10 carbons) and branched (orange) ligands. Data for ligands with an even number of carbons in the backbone are marked with a black circle.

Both the branching position and branching group length effects discussed above included two sets of ligands, which differ from each other not only by the desired effects but also by the backbone length. In order to isolate the effect of the backbone length, we exchanged the native oleate ligands with three alkylthiols of varying backbone lengths; all of them are methyl branched at the fourth carbon position: 4-methyl-1-pentanethiol, 4-methyl-1-octanethiol, and 4-methyl-1-nonanethiol (4-MPT, 4-MOT, and 4-MNT, respectively, Figure 4a). These ligand exchange reactions were compared to *n*-alkylthiols with a similar total number of carbons: 1-hexanethiol, 1-nonanethiol, and 1-decanethiol (HexT, NT, and DT respectively) as references.

The extracted thermodynamic parameters for the facet surface sites are presented in Figure 4 (parameters for the edges can be found at Tables S5 and S6). First, we notice that the observed trends in the changes of the thermodynamic parameters with increasing backbone length are generally the same for both the branched and linear ligand sets. The reaction becomes more exothermic upon exchanging to longer alkylthiols, either branched or linear, due to the formation of more van der Waals interactions (Figure 4b, blue/orange columns represent the linear/branched ligands, respectively). Additionally, as the ligands tend to pack upon binding to the surface, the longer the ligand, the more degrees of freedom are lost, resulting in a more significant entropy loss (Figure 4c). Those trends are consistent with the observations concerning the ligand length dependence in our previous study.^47^

While the overall trends are similar, branching of the chain still affects the measured values of the thermodynamic parameters. We observed a lower enthalpy gain for all three branched ligands compared to that obtained for the corresponding linear ligands. This is consistent with a reduction in the number of van der Waals interactions as a result of steric hindrance associated with the branching methyl group. The same steric hindrance has the opposite effect on the entropy, as it prevents packing of the ligands and lowers the degree of order in the system. This effect is manifested by the pronounced decrease in the entropy loss, measured for branched ligands relative to linear chains of the corresponding total number of carbons.

Comparing the addition of a methyl group to the backbone or as a branching group, while they are identical in terms of the total number of carbons, we reveal two opposite trends concerning the thermodynamics of the system. While linear elongation leads to increased exothermicity and entropy loss, branching lowers the exothermicity and reduces the entropy loss. Furthermore, comparing the 2-MHT and 2-MBT ligands (Figure 2), an additional set that vary in the ligand length, reveals similar behavior, in which the loss of entropy increases with increasing backbone length. However, in this set, the differences in entropy and enthalpy are less pronounced, probably due to the location of the branching position, and the relatively short ligand length. Both are preventing substantial increase/decrease in the exothermicity/entropy loss, respectively, arising from additional van der Waals interactions between the additional two carbons added to the tail of the 2-MHT ligand.

We can use these insights to better understand the interplay between the branching position, the branching group length, and the backbone length, as observed for the 2-MHT/2-EHT and the 4-MOT/4-EOT ligands (Figure 3). Both groups of ligands differ in their backbone length and branching position. With regard to the effect of the branching position, according to the previously mentioned work of Poger *et al*.,^69^ one might think that the 4-MOT ligand should demonstrate a significant lower exothermicity and lower entropy loss, as the branching group is located in the middle of the chain. Indeed the difference in the entropy from the corresponding linear chain is slightly higher for 4-MOT than that of 2-MHT (ΔΔ*S* = 17 and 14 J/molK, respectively), yet the “entropic nature” (characterized by low entropy loss) is similar (Δ*S* = −55 and −54 J/molK, respectively). We suggest that this is due to the lengthening of the backbone, which shields the effect of the change in the branching position. The additional two carbons in the backbone (for 4-MOT), which lead to increased entropy loss (“backbone length effect”), restrain the expected reduction in the entropy loss due to the branching position. For the 2-EHT and the 4-EOT ligands, we observed both a higher difference in the entropy from the corresponding linear chain (ΔΔ*S* = 34 and 3 J/molK, respectively) and also a higher “entropic nature” (Δ*S* = −31 and −66 J/molK, respectively) for the 2-EHT ligand. Recapping, it seems that, for methyl branching ligand, the backbone length effect is stronger than the branching position effect, while for ethyl branched ligands, the interplay between the trends is leaning even more toward the backbone length effect. This interplay will be further elaborated below in the section discussing our theoretical work.

Going back to the backbone length effect in the 4-methyl branched alkylthiols, the opposing trends of increasing exothermicity and decreasing entropy loss result in a minor changes in the Gibbs free energy (Figure 4d). This enthalpy entropy compensation (EEC) is often characterized by a linear relation between Δ*H* and Δ*S*, in which the slope is the compensation temperature (*T*_comp_). This phenomenon was already observed in various biological and chemical systems^70,71^ and also for ligand exchange reactions with linear alkylthiols on Au and on CdSe NCs.^43,47^ For both studied sets, linear (backbone lengths in the range 4–10 carbons) and branched ligands (backbone lengths of 5, 8, and 9 carbons), the compensation temperature (*T*_comp_ = 320 ± 7 and 330 ± 10, respectively) is in good agreement with the experimental temperature (*T*_exp_ = 323), which points to a strong compensation behavior between Δ*H* and Δ*S* (Figure 4e).

Another interesting phenomenon, which was observed within the linear ligands set, is the appearance of an odd–even effect depending on the number of total carbons. When increasing the chain length by one carbon (from an odd number of carbons to an even number or vice versa), opposite trends are observed regarding the decreasing reaction enthalpy and entropy loss. The odd–even effect in regards to surface packing of linear ligands is reported for self-assembled monolayers on flat substrates^72,73^ and also for Au NCs.^74,75^ Despite the generality of this phenomenon in the pure solvent properties^76^ and monolayered surfaces, the origin of this effect is still unclear. Our insights on this effect, including theoretical work using the methodology described below, are discussed further below.

## Determining Conformational Entropy

Greater insight and understanding are garnered *via* theoretical work. In particular, we focus on calculations of the conformational entropy change upon ligand binding in order to both assess the contribution of ligand organization to the overall entropy change of the system and to disentangle the influence of each structural parameter, including backbone length, branching position, and branching group length. To this end, we implemented a mean-field theory previously developed and applied to the self-assembly of lipid chains into micelles and membranes, as well as to grafted polymer brushes.^63−66,77,78^ The theory allows us to determine the probability distribution function (PDF) of the accessible conformations of free and NC-bound ligand given an enumeration of all their possible single-ligand conformations and knowledge of the packing constraints of chains attached to the NC.

To implement this model for the special case of the NC ligands layer, we first calculated the conformational entropy of a linear *n*-carbons length alkylthiol chain with the general structure CH_3_(CH_2_)_*n*−1_-SH. The conformations of the free chain were calculated on the basis of the rotational isomeric state model.^79^ Considering free (unconstrained) rotation of the chain (as expected when the ligand is free in solution) resulted in multiple energetically degenerated configurations. All possible configurational states of the chain were included in calculating the conformational entropy of the free chain, using the known expressions for the canonical ensemble entropy (see SI Section 7.1 for details).

**Figure 5:**
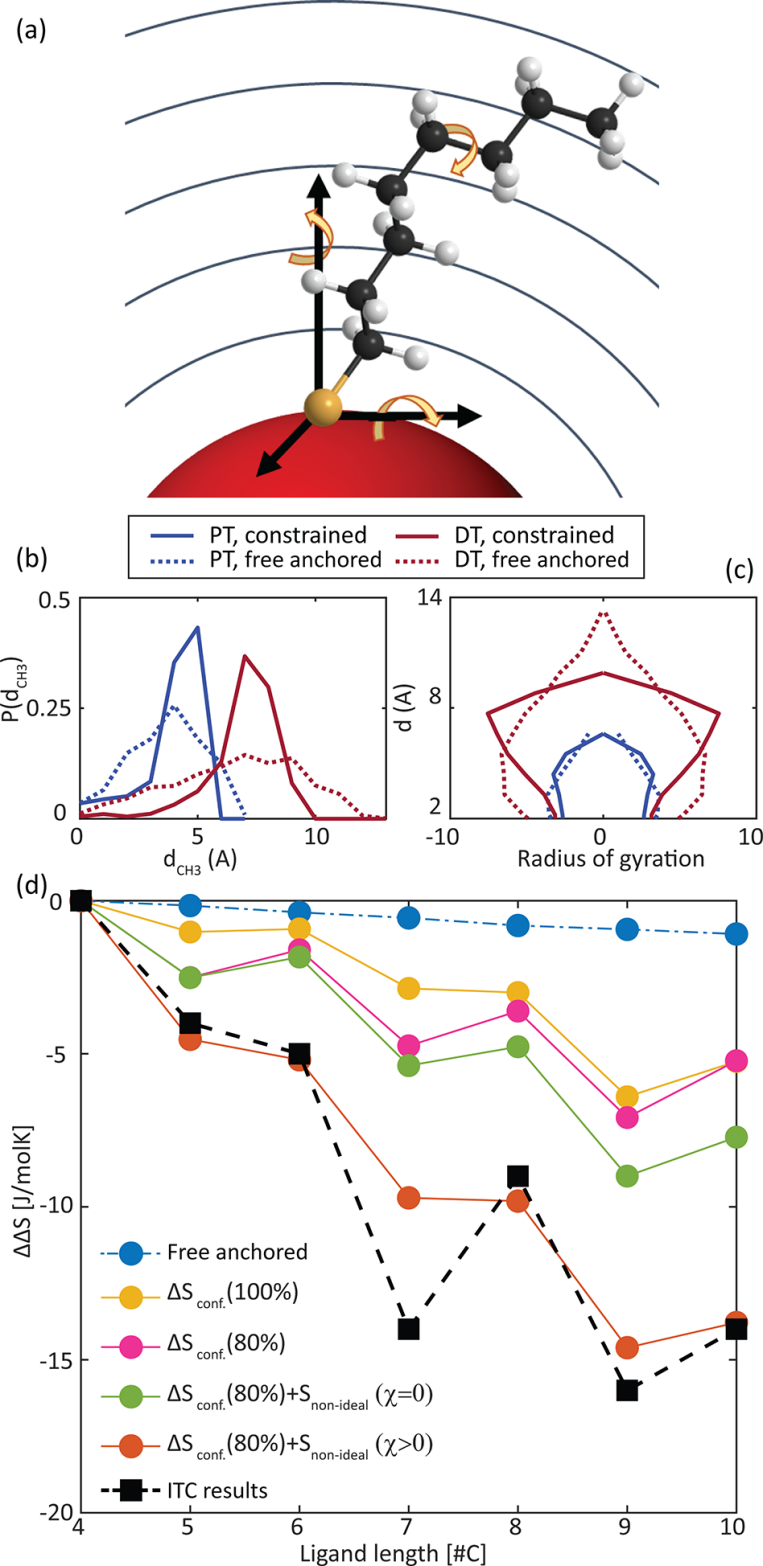
Conformational entropy from model calculations for linear alkylthiols. (a) Illustration of the NC-bound alkylthiolate with the available rotational degrees of freedom (yellow arrows). In the model, the ligand layer volume is divided into several shells and to each layer a packing (density) constraint can be assigned (gray lines, see Section 7.2.2 of the SI). (b and c) Representative simulation results for PT (blue) and DT (dark red) ligands that are free anchored (dashed line) or under a uniform density constraint (solid line). (b) Probability distribution for the distance of the terminal CH_3_ group from the NC surface. (c) Radius of gyration for bound ligand. (d) Experimental ITC results (squares) and calculated conformational entropy changes upon ligand binding shown for: free anchored ligand (blue), constrained ligand on full (yellow), and partially (pink) covered NC, and constrained ligand on partially covered NC considering mixing entropy without (green) and with (brown) Flory interaction parameter χ.

We next enumerated the allowed states for a ligand bound to a spherical NC (Figure 5a). As a starting point, we compared between two extreme packing conditions: (1) a fully constrained, frozen bound ligand that loses its entire conformational entropy upon binding from solution and (2) an anchored ligand, which is not subjected to any constraint on the allowed states (conformations or orientations) as long as it does not overlap (or penetrate) the NC core, which we termed a “free anchored ligand”. The entropy of the bound ligand in the first case is zero, while the entropy of the second is simply derived from all the accessible states and their probabilities (Section 7.2.1 of the SI).

In both cases, calculating the entropy change upon binding for a homologous series ranging in chain lengths between 4 and 10 carbons (the relevant ligand lengths in this study) reveals the experimentally observed trend of increasing entropy loss with increasing chain length (Figure S13). In order to compare the empirical results (for the main binding site) and the calculated conformation entropy, we report entropy differences for all chains with reference to the shortest BT chain (ΔΔ*S* = Δ*S*_RSH_ – Δ*S*_BT_). By doing so, we eliminate the entropy contribution of the bound oleate and the free oleic acid and consider only the net changes upon increasing chain length. Both calculated ligand states exhibit a monotonic entropy change, and as expected, the length dependence is much more pronounced for the frozen bound ligand condition (up to −40 J/molK between BT and DT) than for the free anchored ligand (up to only −1 J/molK between BT and DT). Perhaps not surprisingly, the experimental values for the entropy differences are between those of both conditions (up to −16 J/molK between BT and NT), suggesting that the ligand has some intermediate restriction on the NC surface that is intermediate between the free-anchored and frozen cases.

To include more realistic constraints on chain conformations, we next considered a packing constraint on the ligand shell that is similar to that implemented previously for lipids in membranes.^77^ This constraint sets a defined density of hydrocarbon chains in the ligand shell that matches the density of liquid alkylthiol hydrocarbons (Figure S14). The free energy of the system is then minimized with respect to the applied packing constraint, resulting in a probability distribution of all possible configurations. This allows us to determine all relevant average structural and thermodynamic properties of the ligand chain within the shell. Full details of the calculations are in Section 7.2.2 of the SI.

In comparison to the free anchored ligand model, we find a “compression” of the constrained bound chain toward the NC surface. This is evident from the probability distribution for the location of the terminal CH_3_ group (Figure 5b), which is more confined to smaller distances for the constrained chain (solid line), while in the case of the free anchored ligand, the CH_3_ group is more widely spread away from the NC surface (dashed line). In addition, the radius of gyration (eq S42), which indicates the swelling or expansion of the chain from the normal to the NC surface,^77^ is slightly larger for the constrained long chain relative to the free anchored chain, as the chain tends to “collapse” toward the surface to fulfill the packing constraint explained above (Figure 5c).

The entropy of the constrained bound ligand was calculated using the extracted PDFs assuming full coverage of alkylthiols on the NCs surface (Figure 5d, yellow). Several works reported a double layer structure of capping ligands in nonpolar media,^80,81^ as well as free ligands in diluted samples.^82^ However, the surface analysis for the purified NCs used in this study, before and after ligand exchange, indicated the exclusive presence of bound ligands, with up to 100% surface coverage in total (Section 6 of the SI). Therefore, in order to simulate the experimental conditions, only a single layer of capping ligands was considered in the final NC state. Similar to the experimental results (Figure 5d, black), the entropy loss increases with increasing chain length. Moreover, the conformational entropy calculations also reproduce the “zigzag” seen in the experimental results. Generally, this “odd–even effect” describes oscillations in structure or properties that depend on the presence of either an odd or even number of repeating units, in our case, the number of CH_2_ groups in an alkyl chain. This effect is found in macroscopic material properties, such as the boiling point of liquid *n*-alkanes, and has also been widely observed for properties of chains at various organic/solid surfaces and interfaces. An underlying reason for this effect is that, depending on the even or odd number of CH_2_ units along the alkyl chains, the terminal CH_3_ groups may adopt different orientations, and this in turn affects the packing of the chains within the monolayer.^72^ As evidenced by our ITC experiments, differences in ligand packing and organization on the NCs surface directly impact the thermodynamics of the ligand exchange reactions. Here, we directly observe in the calculation how the constraint on ligand packing in the vicinity of its neighbors results in different entropy changes for the odd vs even chains (Figure S16). The odd–even effect is observed only for the constrained chain and not for the free anchored ligands (or the frozen chain), indicating that this effect arises from the interactions and packing of the ligands with each other rather than from surface binding itself. Moreover, the applied constraint closely corresponds to the “bad solvent” regime, where little or no solvent molecules penetrate into the ligand layer, again supporting the indication that the odd–even effect originates from interligand interactions. By contrast, in the “good solvent” regime, solvent molecules can interpenetrate the ligand layer with no energetic penalty. By applying the appropriate constraint for the good solvent regime in the model,^78^ we find that, in this regime, although entropy differences upon surface binding maintain the same range of values and the entropy monotonically decreases, the appearance of an odd–even effect is suppressed (Figures S15 and S16). This is because solvent molecules that freely penetrate the ligand layer can interrupt the ligands’ close interactions and thus weaken the significant packing constraints. The significant contribution of the ligand packing constraint to the calculated conformational entropy joins previous reports on the ligand shell organization as a crucial parameter for determining NCs colloidal stability,^7−11^ as well as for their photoluminescence properties.^13,14^

The conformational entropy simulations qualitatively reproduce the emergence of the odd–even effect. However, the ΔΔ*S* trend observed in the experiments is more pronounced. To account for this difference, we introduce additional considerations beyond those included so far, which may explain the difference between the model and the experiments. A first important consideration is the ligand exchange yield. Not all of the oleate ligands are exchanged by the alkylthiols, hence the contribution of the alkylthiols to the surface coverage and entropy may be only partial. Thus, on the basis of the average exchange yield measured by TGA, we calculated the entropy change for a lower coverage of alkylthiol (80%) (Figures S10 and S11 and Table S7). Indeed, using this lower value of the coverage, the calculated entropy change, along with the “zigzag” behavior, becomes more pronounced and closer to the experimental observations (Figure 5d, pink).

An additional consideration is the contribution to the entropy that arises from nonideal mixing of ligand and solvent in the solution. The main source of nonideal mixing entropy is related to the volume difference between solvent and ligands. While the shorter BT ligand has a similar molecular volume to the trichloroethylene (TCE) solvent used experimentally, this is not the case for longer ligands. To include this contribution in our calculation, we used the Flory–Huggins theory for mixing, which is commonly applied to polymer melts and solutions.^83,84^ As detailed in the SI (Section 7.2.4), the Flory–Huggins mixing entropy is an approximation that considers only the volume difference between the mixed molecules. In addition, the nonideal Flory–Huggins parameter χ, which represents the change in the overall interactions of the system upon mixing two pure components, can contribute both to the enthalpy and the entropy of the mixing free energy.^85,86^ Thus, the corrected entropy change in our calculation should include both the conformation entropy change and the nonideal mixing between the solvent and the free alkylthiol. Even when setting χ = 0 (Figure 5d, green), our calculation shows a decrease in the calculated entropy affording closer agreement with the experimental results. Dissecting the different contributions to the overall entropy changes, we find that the contribution of the nonideal mixing to the overall entropy is more pronounced for the longer ligands. This is consistent with the origin of the nonideal mixing entropy, which is the volume difference between the ligand and the TCE solvent molecules.

Finally, we consider our observations that ITC dilution experiments of titrating pure alkylthiol to pure TCE showed an exothermic response (Figure S17). This points to nonzero interactions between the alkylthiol and the TCE. As was mentioned, these interactions should also contribute to the entropy with χ > 0, corresponding to the solvent molecule packing with the free alkylthiols. This also justifies considering a ligand length dependence for χ, which decreases with increasing ligand length (Section 7.2.5 of the SI). Such behavior was also observed and confirmed experimentally for polymers.^87−89^ Upon including this consideration, the calculated entropy closely matches the experimental value (Figure 5d, brown). The ability to reproduce the experimental results once these additional terms are considered indicates that, beyond the conformational entropy, additional parameters must be considered to fully account for the entropy change in the NC ligand exchange reaction. Overall, this establishes the strength of this theoretical approach in providing further understanding and insights to the effects governing the thermodynamics of the NC ligands layer and to the different contributions to the system free energy.

**Figure 6:**
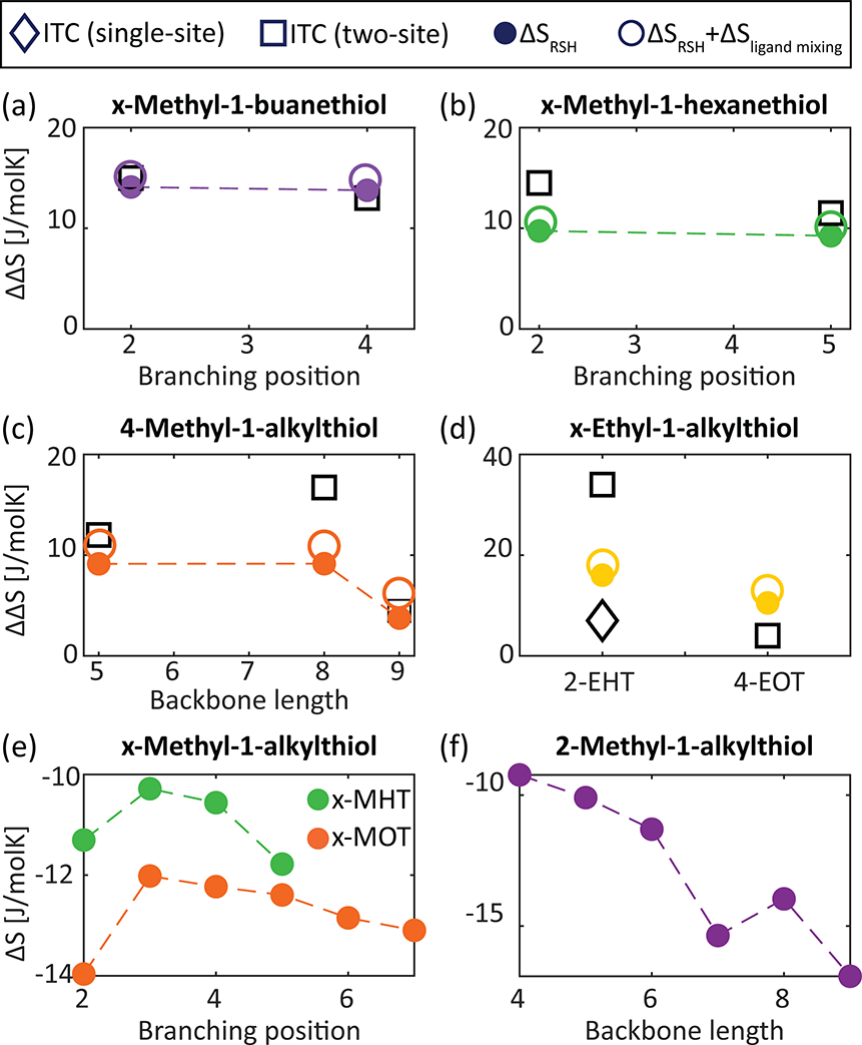
Conformational entropy model calculation for branched alkylthiols. (a and b) Branching position effect in methyl branched (a) butanethiol and (b) hexanethiol. (c) Backbone length effect in 4-methyl branched alkylthiol. (d) Branching position and backbone length effect in ethyl branched alkylthiol. (e) Calculation results for branching position entropy effect in methyl branched hexanethiol (x-MHT, green) and octanethiol (x-MOT, orange). (f) Calculation results for backbone length effect in 2-methyl branched alkylthiol.

With model entropy calculations validated for the linear ligands binding, we proceed next to calculate the entropy change upon branched ligands binding within the same theoretical framework. Figure 6 presents ΔΔ*S* values, taken as the difference between the conformational entropy of bound branched ligands and the corresponding bound linear ligands with the same total number of carbons. Although the experiments were performed with a racemic mixture in the case of chiral branched ligands, for simplicity, the presented calculated entropy change is for a specific enantiomer. Since the volume constraints we imposed are achiral, this has no impact on our calculation, and similar results were observed also for the second enantiomer (Figure S18).

Similar to the analysis for the linear ligands, we used 70% coverage of branched ligands, which is the average exchange yield according to the TGA results (Figure S11 and Table S7). We also considered the nonideality of the ligand–solvent mixing, and the Flory–Huggins interaction parameter was taken to be backbone-length dependent for both the linear and the branched ligands (Figure S17). As can be seen in Figure 6, the calculations mostly reproduce the ITC results of the main binding site, for the investigated branched ligands (full circles). However, 4-MOT exhibits a notable deviation, whereby the experimental data demonstrate a larger difference between the branched and the corresponding linear ligand. As we already concluded for 4-MOT, since the methyl branching is in the middle of the chain, the steric hindrance in ligand packing should be maximal.^69^ The ligand packing in the experimental data are for a racemic mixture, and thus, neighboring opposite enantiomers may exhibit higher steric hindrance and lower organized packing.^90,91^ This effect, which was not included in the calculation that considers a single enantiomer and only achiral packing constraints, may explain the observed deviation. In addition, we notice that, for the 2-EHT ligand, a better correlation between theory and experiment is achieved for parameters extracted from single-site fitting. This is reasonable as no two-site behavior was observed for this ligand, as discussed in the experimental part.

As was observed for the linear ligands, the alkylthiol coverage slightly affects the calculated entropy (Figure 5d, yellow and pink). Indeed, the entropy calculated considering the alkylthiol coverage extracted from TGA measurements mostly reproduces the experimental results. Yet, we further examined the effect of alkylthiol coverage in branched vs linear ligands. Using the same alkylthiol coverage for both linear and branched ligands resulted in a lower entropy difference between the two (Figure S19). Therefore, while the branched ligands still present an intrinsic entropic advantage, the effect of changing the alkylthiol coverage highlights the importance of the ligand shell properties for determining the entropy of the NCs.

An additional contribution to the ligand exchange entropy may be due to the final state of the ligand shell, which contains a mixture of oleate and alkylthiolate. Previously, a mixture of linear ligands was also classified as “entropic ligand”,^61^ and this phenomenon can potentially be generalized to the mixtures of other types of ligands.^14^ The ideal mixing entropy can be closely estimated as the number of states associated with occupying *N*_site_ of Cd^2+^ surface sites by *N*_RS_^*-*^ thiolate and *N*_oleate_ oleate ligands:
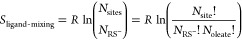
2This contribution cancels out in the ΔΔ*S* presentation for the linear ligands, because a similar average surface coverage was considered for all linear ligands (Figure 5d). However, when comparing 70% coverage of branched ligands with 80% coverage of linear ligands, the entropy of ligand mixing (eq 2) slightly increases the total entropy difference between the branched and the corresponding linear ligand (Figure 6, empty circles). Nonideal mixing entropy can be also considered, which may result from differences in packing of oleate with each of the investigated alkylthiols.^92^ This (probably small) contribution is not included in this study, since the presented model already adequately reproduces the experimentally observed entropy both for linear and branched ligands.

The established calculation framework allows us to expand the study beyond the experimentally accessible branched ligands. Figure 6e presents the binding conformational entropy changes for all available branching positions in alkylthiols with backbone lengths of 6 and 8 carbons (x-MHT and x-MOT, respectively; results for additional chain lengths are presented in Figure S20). Similar to our observations regarding the 2-MHT and the 4-MOT ligands, the backbone length effect dominates the entropy change as the branched hexanethiol ligand exhibits lower entropy loss at almost all branching positions. Generally, as observed experimentally, there is a lower entropy loss for ligands with a branching group located closer to the NC surface. However, the calculations suggest that branching positions located toward the middle of the chain are interestingly the most entropically favorable. A similar phenomenon was observed for phospholipid bilayers, where the disruption due to ligand packing was maximal for chains with methyl branching in the middle position.^69^ Moreover, while for the shorter ligand, the branching in the iso-position seems to be the least entropically favorable, for the longer ligand, this is related to the branching at the second carbon position. It thus seems that the steric hindrance induced for longer ligands by methyl branching located next to the NC surface less affects the end-chain carbons and still allows for partial packing. This suggests again that the entropic effects associated with the backbone lengths are more dominant than the ones associated with the branching position. This inference is also corroborated by the entropy calculations for 2-methyl branching position (Figure 6f, results for additional branching positions are presented in Figure S21). We find that the entropy loss related to the longer ligands increases with increasing chain length, indicating that the branching still allows for considerable ligand–ligand interactions. Additionally, theory predicts a mild odd–even effect for the 2-methyl branching. The trends observed from the calculations suggest that further thermodynamic study of additional branched ligands may reveal insights regarding the ligand shell organization and its contribution to the “entropic” behavior of the branched ligands.

## Conclusions

In summary, combining experimental tools and theoretical modeling, we investigated how structural changes in surface ligands impact the thermodynamics of ligand exchange reactions in CdSe NCs. ITC measurements showed that, in comparison to linear ligands, the reaction becomes less exothermic and involves lower entropy loss for the exchange with branched ligands with a similar number of carbons. This provides direct justification for naming this family of ligands “entropic ligands”. The differences in entropy loss can be attributed to the steric hindrance associated with ligand packing. We gain additional molecular level insight by using mean-field calculations for ligand binding. Our calculations point to the key contribution of the ligand’s conformational entropy changes to the overall change in binding entropy. While we find that the branching position is crucial for ligand packing, the length of the backbone also plays an important role in determining the entropic nature of the ligand. Thus, both parameters—branching position and chain length—should be considered when planning the most “entropic” ligand. Specifically, we find that entropic ligands should correspond to short, branched ligands with a short branching group located toward the middle of the ligand chain. Our results provide insights toward the fundamental understanding of the effect of ligand structure, which should be central for rational planning of NC surface manipulations. Beyond the fundamental understanding, this bears a clear relevance to the application of semiconductor NCs in diverse technologies requiring and utilizing the flexible surface chemistry for compatibility with different solvents and matrixes.

## Materials and Methods

### Chemicals

1-Octadecene (90%), oleic acid (90%), CdO (≥99.99%), Se powder (100 mesh, 99.99%), trichloroethylene (anhydrous, ≥ 99%), 1-butanethiol (99%), 1-pentanethiol (98%), 1-hexanethiol (97%), 1-heptanethiol (98%), 1-octanethiol (≥98.5%), 1-nonanethiol (98%), 1-decanethiol (96%), 2-methyl-1-butanethiol (99%), 3-methyl-1-butanethiol (97%), 2-ethyl-1-hexanethiol (97%), 2-methyl-1-hexanoic acid (98%), 5-methyl-1-hexanoic acid (98%), 4-methyl-1-octanoic acid (98%), 4-ethyl-1-octanoic acid (98%), 4-methyl-1-nonanoic acid (99%), LiAlH_4_, *p*-toluenesulfonyl chloride (99%), thiourea (99%), toluene-*d*_8_ (99.6% D), and chloroform-*d*_1_ (99.8% D) were purchased from Sigma-Aldrich, Alfa Aesar, or Acros Organics.

### Methods

Absorption measurements were performed using a JASCO V-770 UV–vis–NIR spectrophotometer. NMR measurements were performed using a Bruker 400 MHz NEO 5 mm BBFO probe. ITC experiments were performed using a NanoITC calorimeter (TA Instruments) equipped with a 1 mL Hastelloy sample and reference cells with a 250 μL syringe. Thermogravimetric analysis (TGA) measurements were performed using a TGA-5500 (TA Instruments). Attenuated total reflection Fourier transform infrared (ATR-FTIR) measurements were performed using a Nicolet iS50 FT-IR spectrometer with Smart iTX accessory (Thermo Scientific). ITC curves fitting and conformational entropy calculations were done using MATLAB (The MathWorks Inc.).

### CdSe NCs Synthesis

CdSe NCs (*d* = 3 nm) in a zinc blende structure were synthesized by modifying a known procedure.^14^ Briefly, in a 100 mL three-neck flask, 4 mL of 0.2 M Cd-oleate and 13 mL of ODE were degassed under a vacuum at 100 °C for 1 h. Then, the temperature was increased to 240 °C under Ar flow and 4 mL of 0.1 M Se suspension in ODE was quickly injected. Additional 200 μL of 0.1 M Se-ODE suspension was injected every 5 min to avoid Ostwald ripening. The NCs reached the desired size (*d* = 3 nm) after 30 min. The NCs were precipitated from the synthesis crude solution by centrifugation with toluene and ethanol at 60K RPM for 10 min. Then, the NCs were redispersed in toluene and precipitated again with ethanol. The last step was repeated three times in order to get rid of excess ligands. The NCs clean solution was kept in trichloroethylene (TCE) under an Ar atmosphere. The NC size was determined from a previously reported sizing curve on the basis of the first exciton peak position and correlated with the size measured from transmission electron microscopy (TEM) images of the sample (Figure S1).^93,94^

### Synthesis of Branched Alkylthiols

Commercially available branched chain carboxylic acids were converted to the corresponding thiols *via* a three-step procedure adapted from known protocols (Scheme S1).^95,96^ A detailed procedure is mentioned in Section 3 of the SI. Briefly, a reduction of the relevant carboxylic acid into the corresponding alcohol was performed. Following that, the alcohol was reacted with *p*-toluenesulfonyl chloride to give a tosylated product, which was later reacted with thiourea to yield the desired thiol derivative. After every step, NMR spectra of organic substances were recorded, to determine the structure and composition of the organic products. An exemplary analysis of the synthesis of the branched ligands is given on 4-MNT in Figure S2. ^1^H NMR spectra of the final product are presented in Figure S3.

### ITC Measurements

Alkylthiol ligands and purified NCs dispersed in TCE were used for ITC measurements. This solvent was chosen due to its relatively high boiling point and a relatively low enthalpy of mixing with the ligands.^97^ The NCs concentration was determined from the solution absorption, on the basis of a previous report of the extinction coefficient.^93^ The surface sites concentration was calculated on the basis of a simple spherical model of zinc blende CdSe with a lattice parameter of 6.050 Å (see Section 4 of the SI). For each titration, 1 mL of NCs solution was injected to the ITC sample cell and the ligand solution was loaded in the 250 μL ITC syringe. The surface sites and the ligands concentration were adjusted in order to produce high quality titration curves. At each injection step, 5 μL of ligands solution was injected to the cell and the heat flow was measured for 600–800 s during which the system returned to equilibrium. All ITC thermograms and exchange-model fitted titration curves, including detailed derivation of the single-site and two-site models, are presented in Section 5 of the SI.

### Conformational Entropy of Free and NC-Bound Alkylthiol

All calculations were done considering an alkylthiol chain with a C–S bond length of 1.82 Å and a C–C bond length of 1.54 Å. Ligand conformations were determined according to the rotational isomeric state model,^79^ considering *trans* (*t*, dihedral angle of 0°) and *gauche* (*g*^*+*^ and *g*^*-*^, dihedral angles of +120° and −120°, respectively) conformations for the relevant bonds. For each conformation, several orientations, resulting from free rotation of the chain in space, were considered. For free and NC-bound ligands, the conformational entropy was calculated by enumerating the allowed states, subjected to the chosen constraints, as detailed in the main text and also in Section 7 of the SI.

## Supporting Information Available

Figures of NCs absorption spectra, TEM images, and size distribution histogram, discussions of *n*-alkylthiols reduction, synthesis of branched alkylthiols, including scheme of synthesis, Figures of ^1^H NMR spectra recorded for the products, and Table of final composition of the obtained products, surface sites calculation and Figure of the simulated atomistic model, ITC experimental data and analysis includings Figures of real-time thermograms and the corresponding titration curves and fits, and Tables of the thermodynamic parameters extracted from the single- and two-site model fit, additional surface characterization: FTIR and TGA including Figures of FTIR spectra andTGA thermograms before and after ligand exchange, and Table of post-ITC ligand composition, calculation results and discussion for free and NC-bound linear and branched alkylthiols, including Figures of conformational entropy, order parameter of the terminal bond and odd-even effect for surface bound alkylthiol, surface coverage effect on the overall entropy difference between branched and linear ligands, and ITC measured mixing heat for alkylthiol and TCE (PDF)
