# Supplementary material for: Entropy of Branching Out: Linear versus Branched Alkylthiols Ligands on CdSe Nanocrystals
Source: ACS Nano. 2022 Feb 14;16(3):4308–21. doi: 10.1021/acsnano.1c10430 (PMC8945696; doi:10.1021/acsnano.1c10430)
Supplement: Supplementary file 1 — nn1c10430_si_001.pdf [file nn1c10430_si_001.pdf]

Supporting Information

Entropy of Branching Out: Linear versus Branched Alkylthiols Ligands on CdSe Nanocrystals

Orian Elimelech,[a] Omer Aviv,[a] Meirav Oded,[a] Xiaogang Peng,[b] Daniel Harries,[a,c] and Uri Banin[a]**

a The Institute of Chemistry and The Center for Nanoscience and Nanotechnology, The Hebrew University of Jerusalem, Jerusalem 9190401, Israel.

b Department of Chemistry, Zhejiang University Hangzhou, 310027 P. R. China

c The Fritz Haber Center, The Hebrew University of Jerusalem, Jerusalem 9190401, Israel.

*E-mail: Uri.Banin@mail.huji.ac.il

Daniel.Harries@mail.huji.ac.il

Table of contents:

1. **CdSe NCs synthesis** 3
2. **n-Alkylthiols reduction** 3
3. **Synthesis of branched alkylthiols** 4
4. **Surface sites calculation** 8
5. **ITC measurements and analysis** 10
   5.1. Derivation of a single-site ligand exchange model 10
   5.2. Derivation of a two-site ligand exchange model 12
6. **Additional surface characterization: FTIR and TGA** 21
7. **Conformational entropy calculation** 26
   7.1. Conformational entropy of free ligands 26
   7.2. Conformational entropy of NC-bound ligands 27

1. CdSe NCs synthesis

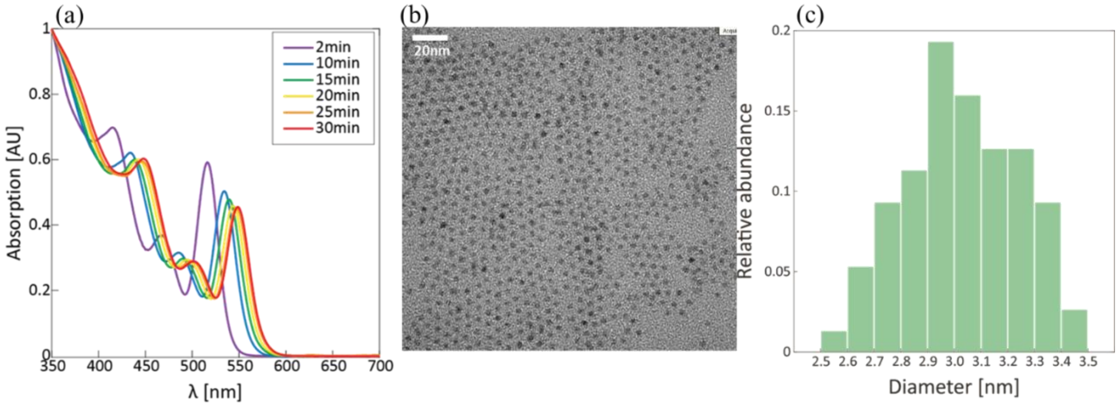

Figure S1. *(a) Absorption spectra of aliquots during a typical oleate coated CdSe NCs synthesis (b) TEM image of oleate coated CdSe NCs, (c) Size distribution histogram for oleate coated CdSe NCs, the average diameter is 3.0±0.2 nm.*

2. n-Alkylthiols reduction

As discussed in detailed in our previous study,[1] in order to avoid inaccuracies in the ligands concentration derived by S-S chain coupling bonds, all purchased ligands were reduced prior to use. Shortly, the reduction was done by adding two equivalents of $NaBH_4$ powder to a solution of alkylthiol in ethanol and TDW (1:4). After 12 hours of stirring at room temperature, extraction was performed with chloroform (3x75 ml portions). Following that, the unified organic phase was dried over $MgSO_4$ and then filtered and evaporated under vacuum in order to separate between the chloroform and the reduced alkylthiol. The reduced ligands were kept under an inert atmosphere with no exposure to UV light for future use.

As was reported previously,[1] the yield of the reduction procedure is 70%, to give a final product with only about 5% of disulfide.

3. Synthesis of branched alkylthiols

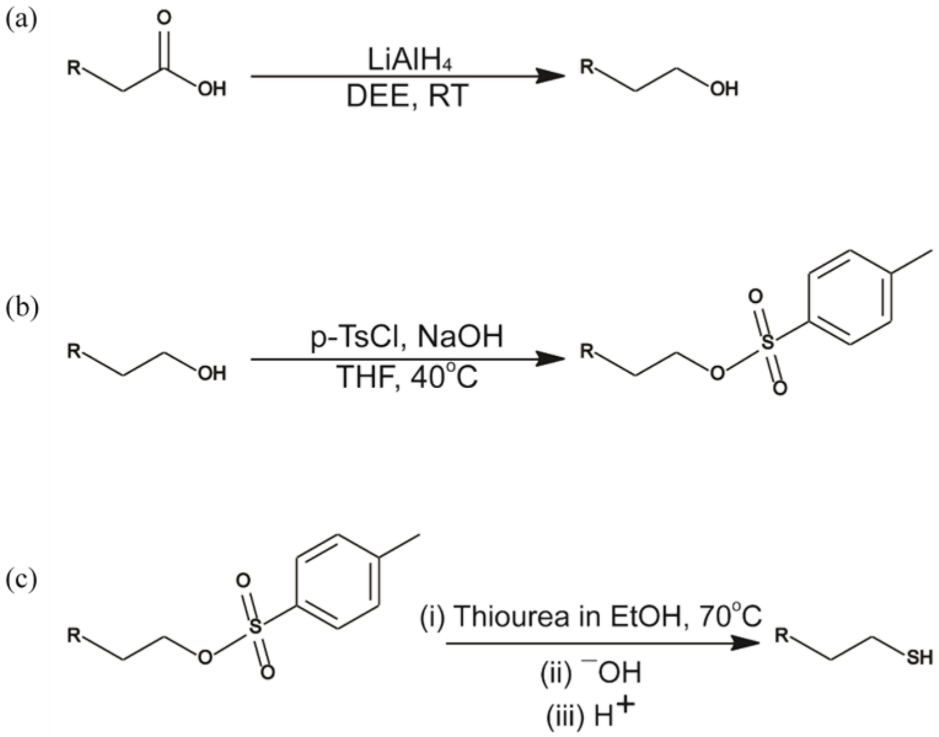

Scheme S1. *Synthesis of branched alkylthiols: (a) Reduction of carboxylic acid to alcohol. (b) Tosylation of primary alcohol. (c) Thiolation of tosyl-alcohol.*

- ***Step 1*– *carboxylic acid reduction (Scheme S1a)*[2]**

200 ml of diethyl-ether (DEE) were loaded into a three-necked flask. Under Ar flow, $LiAlH_4$ (200 mmol) was added into the flask, and the mixture was stirred for 10 minutes at RT. A solution of branched chain carboxylic acid (50 mmol) in 40 ml DEE was added dropwise over a period of 1h. The reaction mixture was stirred overnight, and then quenched carefully with HCl (600 mmol), followed by extraction with DEE (3x75 ml). The organic phase was dried over $MgSO_4$, filtered, and the solvent was evaporated under vacuum to give the branched chain alcohol.

Figure S2 and S2b presents the ^{1}H-NMR spectra of the 4-methylnonanoic acid and 4-methyl-1-nonaol (red and blue, respectively). The broad peak attributed to the COO**H** acidic proton (inset, δ=11.2 ppm) disappears after the reduction process, while we notice the appearance of a broad peak at δ=1.46 ppm that is assigned to the O**H** proton, as well as a triplet at δ=3.61-3.64 ppm that is related to the C$\mathbf{H}_2$ group adjacent to the OH head group. The full disappearance of the acidic proton indicates the complete conversion of the process.

- ***Step 2*– *tosylation of primary alcohol (Scheme S1b)*[3]**

`

In a 150 ml three-neck flask, 135mmol NaOH were dissolved in 12 ml TDW. Branched chain alcohol (50 mmol) dissolved in 25 ml of THF was added to the flask. The mixture was stirred at RT for 5 minutes, and then cooled down to 0°C. Under Ar flow, a solution of p-TsCl (55 mmol) in 30 ml THF was added dropwise, over 1 hour. The flask was then heated to 35°-C, and the reaction proceeded for 72 hours. After cooling back to RT, the reaction mixture was extracted with DEE (3x75 ml portions), followed by the extraction of the organic phase with 20 ml portions of TDW, until the aqueous phase was neutralized. The organic phase was dried over $MgSO_4$, filtered, and the solvent was evaporated under vacuum.

^{1}H-NMR spectrum (Figure S2, green) of the tosylated product shows a down field shift of the C**H**$_2$ related triplet (δ=3.99-4.02 ppm). This effect is due to the presence of the tosyl (electron withdrawing) group in close proximity to the C**H**$_2$ group. Additional peaks are assigned to the aromatic protons (doublet at δ=7.78-7.80 ppm, doublet at δ=7.33-7.35 ppm), and to protons of the C**H**$_3$ group on the aromatic ring (δ=2.45 ppm).

- ***Step 3*** *– thiolation of tosyl-alcohol (Scheme S1c)*[3]

100 ml of dry EtOH were loaded into a 500 ml flask under Ar atmosphere along with tosylated alcohol (50 mmol). The solution was stirred for 10 minutes, followed by the addition of thiourea (100 mmol). The reaction mixture was refluxed at 70°C for 72 hours, then 5 M NaOH solution (85 ml) was added slowly, and the solution was refluxed for additional 4 hours. The flask was cooled to RT, and the reaction mixture was carefully acidified with 1 M HCl to reach pH=5, followed by extraction with DCM (3x75 ml portions). The organic phase was dried over $MgSO_4$, filtered, and evaporated under vacuum to obtain the desired branched chain alkylthiol.

According to the ^{1}H-NMR spectrum (Figure S2, purple), the obtained product is a mixture containing the desired methyl branched alkylthiol (C**H**$_2$-SH quartet, δ=2.48-2.53 ppm, S**H** triplet, δ=1.32-1.36 ppm), and the corresponding primary alcohol (C**H**$_2$-OH triplet, δ=3.61-3.64 ppm). Also, a triplet at δ=2.65-2.69 ppm indicates the formation of disulfide molecules, caused by oxidation of the thiols. Since according to the ^{1}H-NMR spectra the tosylation of the primary alcohol is complete, it appears that only a part of the tosylated intermediates reacted to give the thiolated product, while the unreacted portion transformed back into alcohol. We calculated the ratio between integrated areas of C**H**$_2$-OH/C**H**$_2$-SH/C**H**$_2$-S-S-C**H**$_2$ peaks, in order to accurately calculate the alkylthiol concentration used for every ITC experiment. Short-chain alkylthiols were extremely volatile, preventing the complete evaporation of ethanol residues. The presence of ethanol in the final products was also taken into account, as

will be discussed below. Figure S3 presents the NMR spectra recorded for the final products, and the final compositions of all synthesized ligands are summarized in Table S1.

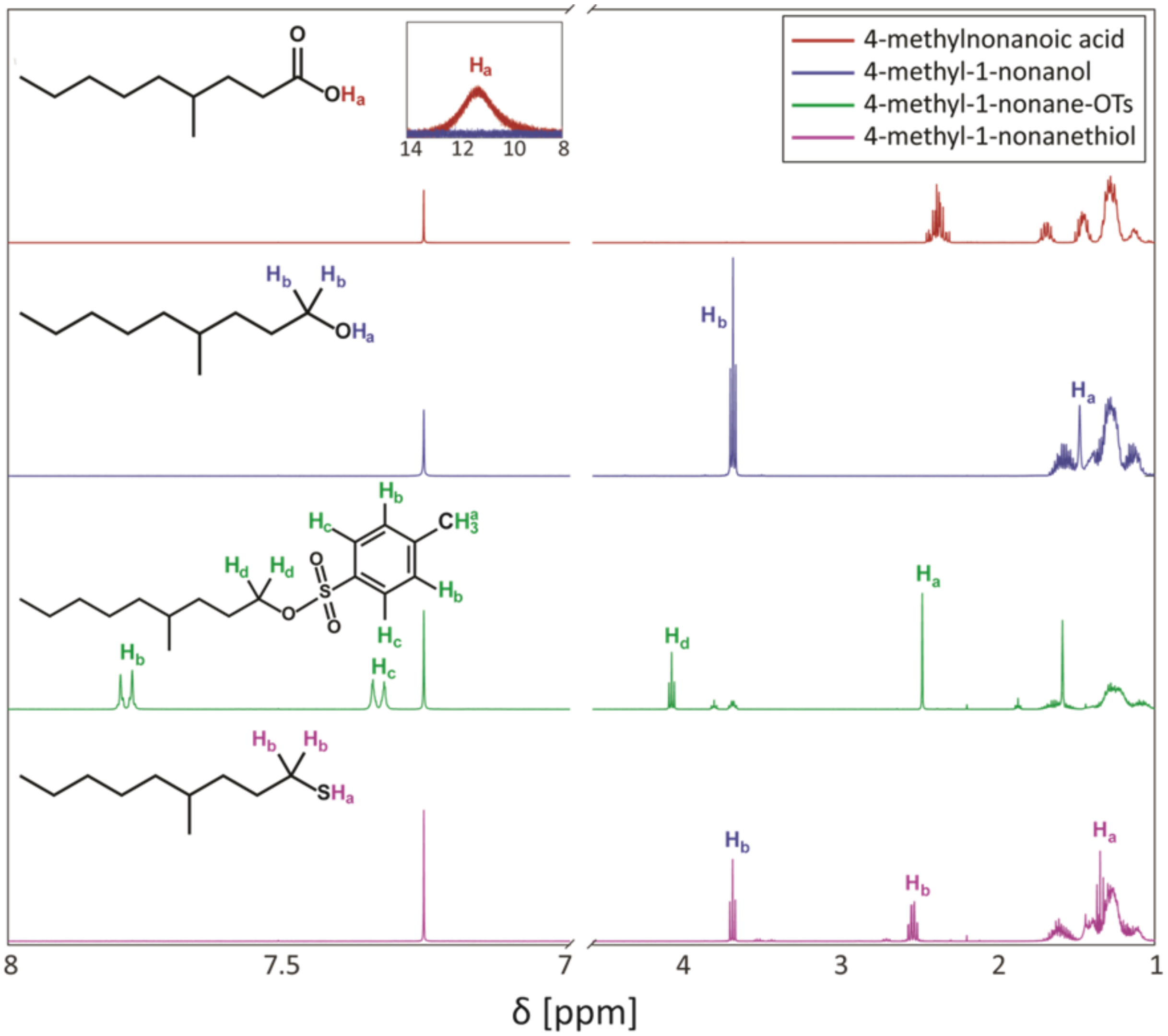

Figure S2. *^{1}H-NMR spectra in different stages of branched alkylthiol synthesis demonstrated for 4-mehtyl-1-nonanethiol, 4-MNT, as a representative branched alkylthiol: (a) 4-methyl-nonanoic acid (4-MNA, red), (b) 4-methyl-1-nonanol (4-MNOH, blue), (c)4-methyl-1-nonyl tosylate (4-MNOTs, green), (d) 4-methyl-1-nonanethiol (4-MNT, purple). Inset: 8-14ppm region of 4-MNA, 4-MNOH spectra. All spectra were recorded in chloroform-d.*

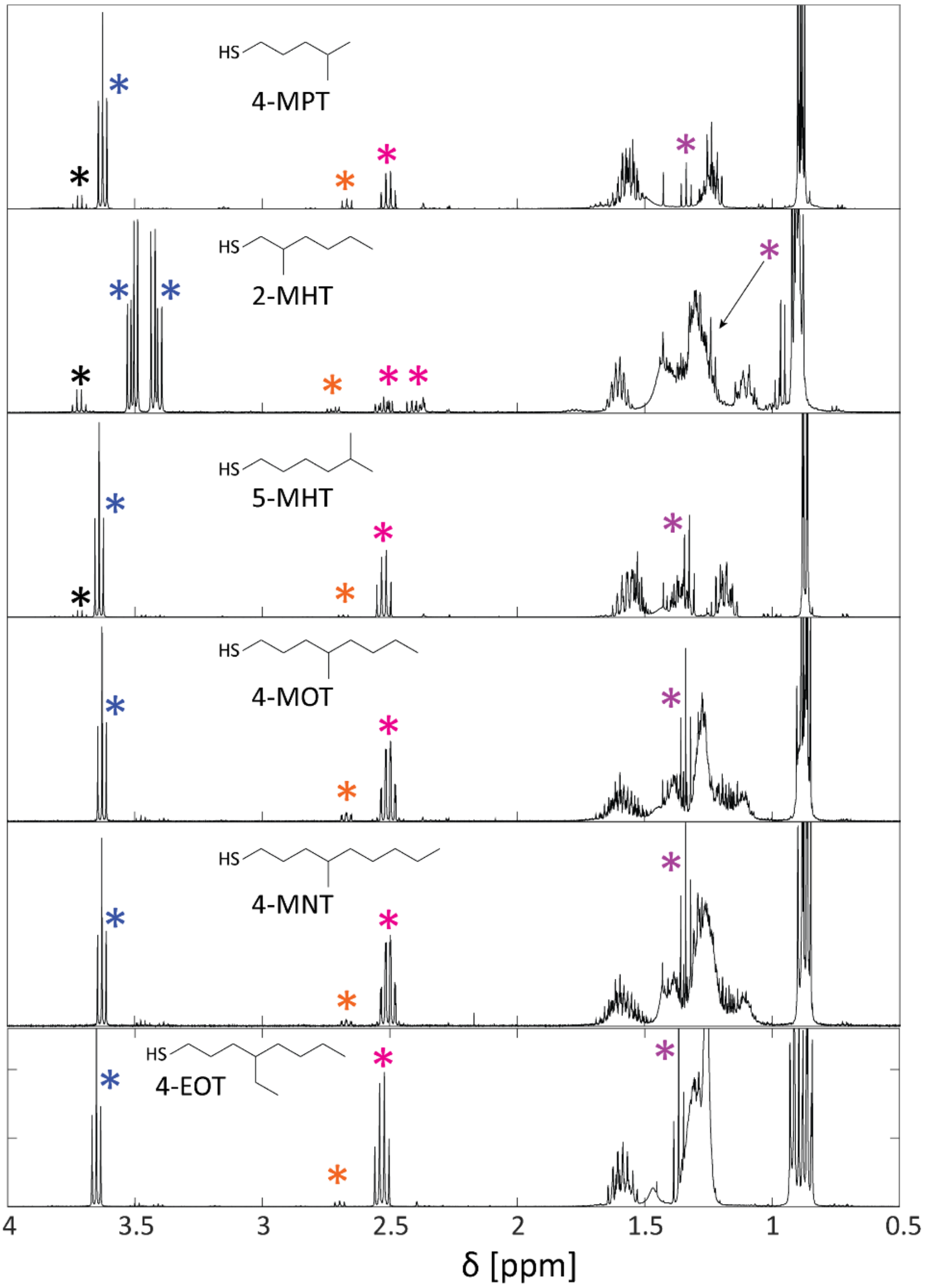

Figure S3. *^{1}H-NMR spectra of organic synthesis final products: (a) 4-methyl-1-pentanethiol (4-MPT), (b) 2-methyl-1-hexanethiol (2-MHT), (c) 5-methyl-1-hexanethiol (5-MHT), (d) 4-methyl-1-octanethiol (4-MOT), (e) 4-methyl-1-nonanethiol (4-MNT) and (f) 4-ethyl-1-octanethiol (4-EOT). All spectra were recorded in chloroform-d. Different components were identified based on the following peaks (marked with asterisks): peaks marked with purple and pink asterisks are assigned to R-SH and* CH_2*-SH (respectively) of the desired alkylthiol molecules, peaks marked with orange asterisk are assigned to R-*CH_2*-S-S-*CH_2*-R of corresponding disulfides, peaks marked with blue asterisks are assigned to R-*CH_2*-OH of corresponding alcohol molecules, and peaks marked with black asterisk are assigned to* CH_3*-*CH_2*-OH of ethanol residues.*

Desired product	R-OH fraction	RS-SR fraction	R-SH fraction	EtOH fraction
***4-methyl-1-pentanethiol* (4-MPT)**	0.67	0.06	0.23	0.04
***2-methyl-1-hexanethiol* (2-MHT)**	0.81	0.02	0.13	0.04
***5-methyl-1-hexanethiol* (5-MHT)**	0.56	0.02	0.40	0.02
***4-methyl-1-octanethiol* (4-MOT)**	0.39	0.06	0.55	-
***4-methyl-1-nonanethiol* (4-MNT)**	0.37	0.04	0.59	-
***4-ethyl-1-octanethiol* (4-EOT)**	0.39	0.03	0.58	-

Table S1. *Final composition of the obtained products, as calculated based on ^{1}H-NMR integration ratios from the spectra presented in Figure S3.*

4. Surface sites calculation

As mentioned in our previous study,[1] the number of Cd surface sites ($N_{surface}$) was calculated based on a simple spherical model for the NCs with a lattice parameter of a=6.050Å (zinc blende). We assumed a uniform zinc blende CdSe layer on the surface, hence the number of Cd surface sites is

(S1) $$N_{surface} = N_{total} - N_{internal-sphere}$$

where N_{total} is the total number of Cd atoms in the NC and $N_{internal\text{-}sphere}$ is the number of core Cd. N_{total} was calculated considering the volume of a spherical NC with a radius R_{NC}, the density of CdSe (ρ_{CdSe}) and its molar mass (Mw_{cdSe}):

(S2) $$N_{total} = \frac{\frac{4}{3}\pi R_{NC}^{3} \cdot \rho_{CdSe}}{Mw_{CdSe}} \cdot N_A$$

$N_{internal\text{-}sphere}$ was calculated in a similar way to N_{total} excluding the outer layer of the surface Cd:

(S3) $$N_{internal-sphere} = \frac{\frac{4}{3}\pi\left(R_{NC}-\frac{a}{2}\right)^{3} \cdot \rho_{CdSe}}{Mw_{CdSe}} \cdot N_A$$

For the investigated d=3.0 nm NC, 127 Cd surface sites are expected.

The results were compared with a pyramidal model for zinc blend CdSe NCs with four exposed (111) facets. The height of the pyramid, *h,* was taken as the calculated diameter of the NC, hence the edge length, *c*, is:

(S4) $$c = \sqrt{\frac{3}{2}}h$$

The Cd atoms are spaced on the edge according to a nearest-neighbor distance, *d*, of the unit cell:

(S5) $$d = \frac{\sqrt{2}}{2}a$$

Hence, the length of an edge, *c*, containing *N* atoms is:

(S6) $$c = \frac{1}{\sqrt{2}}a(N-1)$$

Using eq. (S4) in eq. (S6), we can determine the number of atoms on the edge:

(S7) $$N = \frac{h\sqrt{3}}{a} + 1$$

Since the zinc blend NCs are actually a truncated pyramid, the atoms of the outer edges were removed, and the new faces lost one atoms per line, per side. Therefore, the number of Cd surface atoms on a single face, N_{face}, with a base containing *(N-2)* atoms is calculated by:

(S8) $$N_{face} = \sum_{q=1}^{N-2} q = \frac{(n-2)(n-2+1)}{2}$$

By using eq. (S7) in eq. (S8) and multiplying it by 4 (for the four faces), we find the total number of Cd surface atoms in all four faces as a function of the pyramid height:

(S9) $$N_{surface} = 2\left(\frac{h\sqrt{3}}{a} + 1\right)^2 - 6\left(\frac{h\sqrt{3}}{a} + 1\right) + 4$$

For the investigated d=3.0nm NC, 130 Cd surface sites are expected.

In addition, an atomistic model was also considered to verify the suggested models for surface sites. A semi-spherical NC was simulated from the bulk zinc blende CdSe crystal structure by removing atoms locating beyond a distance which is greater than the desired radius (Figure S4). The remained atoms resulted in a non-stoichiometric ratio between Cd and Se atoms. The atoms in the outer layer were considered as surface sites. Results for NC diameter of 2.8, 3.0 and 3.2 nm are presented in Table S2. For an average d=3.0±0.2 nm NC (similar to the experimentally investigated NCs, Figure S1), 128±47 Cd surface sites are expected with an average Cd:Se ratio of 1.1±0.1. The non-stoichiometric ratio we find is consistent with previous reports on CdSe NCs.[4]

All presented models give similar numbers of Cd surface sites. For simplicity of the ligand shell presentation in section 7, we used the spherical model.

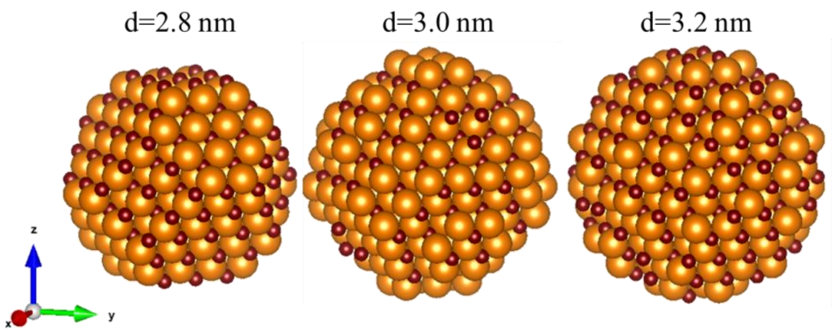

Figure S4. *Simulated atomistic model for zinc blend CdSe NC of diameters 2.8, 3.0 and 3.2 nm. Cd and Se atoms are colored brown and orange respectively.*

NC diameter	Cd atoms	Cd:Se ratio	Surface Cd	Surface Se
**2.8**	201	1.14	114	96
**3.0**	225	0.94	90	100
**3.2**	321	1.16	180	124

Table S2. *Summary of the simulated atomistic model data for zinc blend CdSe NC of diameters 2.8, 3.0 and 3.2 nm, as presented in Figure S4.*

5. ITC measurements and analysis

5.1. Derivation of a single-site ligand exchange model

The ligand exchange model is based on the well-known "single set of independent binding sites" model.[5] We modified the previously used model in order to take into account the detachment of the native ligand and the attachment of the new ligand, in contrasts to the "single set of independent binding sites" model which takes into account only the binding events. The new model is necessary for ligand exchange reactions since both processes, detachment and attachment of the ligands, release heat which is measured by the ITC instrument.

The ligand exchange reaction between the native ligand L' and the exchanged ligand L for a single surface site M is

(S10) $ML' + L \rightleftharpoons ML + L'$

The equilibrium constant is defined as

(S11) $K = \frac{[ML][L']}{[ML'][L]}$

Assuming that each native ligand L' is exchanged with a single new ligand L and no free L' is present initially[6] (supported by TGA data, see in the next section), we get:

(S12) $[ML] = [L']$

`

While for the exchanged ligands:

(S13) $[L] = [L]_0 - [ML]$

And for all surface sites:

(S14) $[ML'] = n[M]_0 - [ML]$

In the previous equations, $[M]_0$ is the total number of surface sites on the NC (based on a spherical model, as explained before), n is the ratio between the actual exchanged ligands and the available surface sites (i.e. the reaction stoichiometry coefficient), and $[L]_0$ is the total added exchanged ligand.

Given the expressions above, the equilibrium constant can be written as:

(S15) $K = \frac{[ML]^2}{(n[M]_0-[ML])([L]_0-[ML])}$

We define θ as the NC surface coverage, and hence,

(S16) $[ML] = n[M]_0\theta.$

Given eq. (S12), eq. (S15) can be rewritten as

(S17) $0 = \theta^2 - \theta\left(\frac{K}{K-1}\right)\left(1 + \frac{[L]_0}{n[M]_0}\right) + \left(\frac{K}{K-1}\right)\left(\frac{[L]_0}{n[M]_0}\right)$

During an ITC experiment, we measure the total amount of heat released per injection of ligand, which is correlated with the enthalpy change of the reaction

(S18) $Q_{total} = \theta n[M]_0 V_{cell}\Delta H$

By using the solution for the quadratic equation(S17), eq. (S18) can be written as

(S19) $Q_{total} =$

$$\frac{n[M]_0V_{cell}\Delta H}{2}\left[\left(\frac{K}{K-1}\right)\left(1 + \frac{[L]_0}{n[M]_0}\right) - \sqrt{\left(\frac{K}{K-1}\right)^2\left(1 + \frac{[L]_0}{n[M]_0}\right)^2 - 4\left(\frac{K}{K-1}\right)\left(\frac{[L]_0}{n[M]_0}\right)}\right]$$

and the heat released per injection of ligand is

(S20) $$\frac{dQ_{tot}}{d[L]_0} = \frac{V_{cell}\Delta H}{2}\left(\frac{K}{K-1}\right)\left[1 - \frac{\frac{[L]_0}{n[M]_0}+\frac{2-K}{K}}{\sqrt{1+\left(\frac{[L]_0}{n[M]_0}\right)^2+\left(\frac{[L]_0}{n[M]_0}\right)\left(\frac{4-2K}{K}\right)}}\right]$$

where

(S21) $d[L]_0 = \frac{V_{injection}[L]_{syringe}}{V_{cell}}$

By implanting eq. (S21) into eq. (S20), we get the final equation for fitting

(S22) $$dQ_{tot} = \frac{V_{injection}[L]_{syringe}\Delta H}{2}\left(\frac{K}{K-1}\right)\left[1 - \frac{\frac{[L]_0}{n[M]_0}+\frac{2-K}{K}}{\sqrt{1+\left(\frac{[L]_0}{n[M]_0}\right)^2+\left(\frac{[L]_0}{n[M]_0}\right)\left(\frac{4-2K}{K}\right)}}\right]$$

`

The other thermodynamics parameters ΔG and ΔS are calculated by using the known thermodynamics relations

(S23) $\Delta G = -RT\, ln\, K$

(S24) $\Delta S = \frac{\Delta H - \Delta G}{T}$

5.2. Derivation of a two-site ligand exchange model

In addition to the above analysis, we also derived a model which considered two different types of surface sites, in agreement with recent studies demonstrating different facets of CdSe NC to have different affinity to the ligands.[7,8] In the model, M_1 and M_2 denote two different independent surface sites bound to the native ligand, L. Hence, the ligand exchange reactions with the new ligand L' for each site are

(S25) $(a)\, M_1L' + L \rightleftharpoons M_1L + L'$ $\qquad (b) M_2L' + L \rightleftharpoons M_2L + L'$

The equilibrium constants are defined as

(S26) $(a)\, K_1 = \frac{[M_1L][L']}{[M_1L'][L]}$ $\qquad (b)\, K_2 = \frac{[M_2L][L']}{[M_2L'][L]}$

Assuming that each native ligand L' is exchanged with a single new ligand L and no free L' is present initially (supported by TGA data, see in the next section), we get:

(S27) $[L'] = [M_1L] + [M_2L]$

While for the exchanged ligands

(S28) $[L] = [L]_0 - [M_1L] - [M_2L]$

where $[L]_0$ denotes the concentration of the overall added ligand L.

And for all surface sites:

(S29) $(a)\, n_1[M]_0 = [M_1L] + [M_1L']$ $\qquad (b)\, n_2[M]_0 = [M_2L] + [M_2L']$

where $[M]_0$ is the total number of surface sites on the NC (based on a spherical model, as explained before), n_i is the ratio between the actual exchanged ligands and the overall available surface sites.

Given the expressions above, eq. (S26) can be solved for $[M_1L]$ and $[M_2L]$ as a function of the fitting parameters n_1, n_2, K_1, K_2 and the known concentrations of surface sites $[M]_0$ and added ligand $[L]_0$.

During an ITC experiment, we measure the total amount of heat released per injection of ligand, which is correlated with the enthalpy change of the reaction:

(S30) $Q_{total} = V_{cell}(\Delta H_1[M_1L] + \Delta H_2[M_2L])$

The fitting equation for this model is given by the derivation of eq. (S30) for the added new ligand $[L]_0$. The other thermodynamics parameters $\Delta G_1, \Delta G_2$ and $\Delta S_1, \Delta S_2$ are calculated by using the known thermodynamics relations (eq. (S23) and (S24)).

Experimental data and fitting

All fitting were done in NanoAnalyze Software v 3.10.0 (TA instrument).

5.3.1. Error analysis:

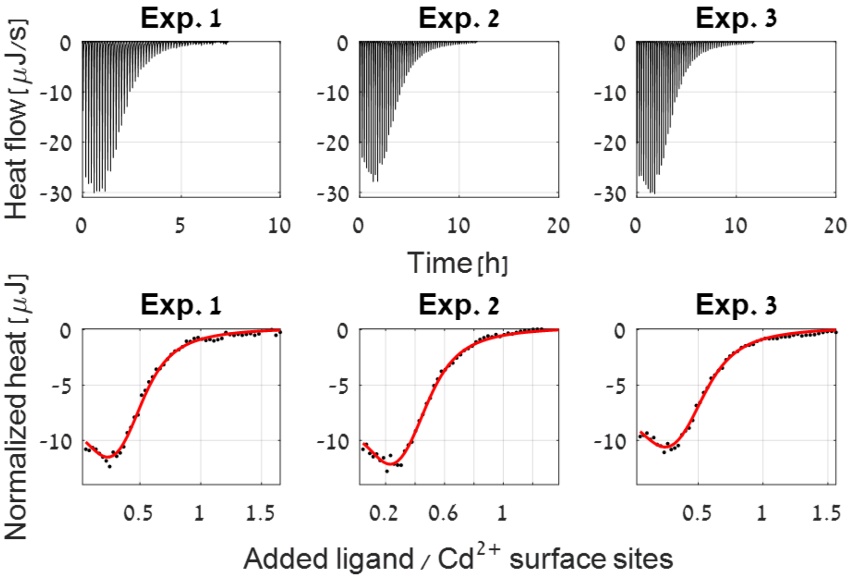

Figure S5. *Real-time thermograms and the corresponding titration curves with their model fitting for the ligand exchange reaction of oleate coated CdSe NCs with 2-methyl-1-butanethiol at 323K.*

	Ligand [mM]	Surface sites [mM]	ΔH [kJ/mol]	K_{eq}	n	ΔS [J/molK]	ΔG [kJ/mol]
**Exp. 1**	30	5.5±0.2	-21.0±0.7	10±1	0.31±0.02	-46±4	-6.1±0.5
			-8.5±0.3	49±7	0.19±0.01	6.0±0.6	-10.4±0.5
**Exp. 2**	25	5.6±0.2	-21.0±0.7	11±1	0.29±0.02	-45±4	-6.5±0.5
			-10.0±0.3	85±10	0.17±0.01	6.0±0.6	-11.9±0.5
**Exp. 3**	30	5.8±0.2	-20.5±0.6	10±1	0.33±0.02	-44±4	-6.3±0.5
			-8.0±0.3	51±7	0.19±0.01	8.0±0.8	-10.6±0.5

Table S3. *The thermodynamic parameters extracted from the model fit of the titration curves for the ligand exchange reaction of oleate coated CdSe NCs with 2-methyl-1-butanethiol at 323K, presented in Figure S5. The presented errors were calculated based on the quality of the fitting.*

Errors of the extracted thermodynamics parameters were determined by the quality of the fitting. In addition, we considered the reproducibility of the measurement by performing the same experiments three times and calculating the standard deviation of each parameter.

The error in the surface sites concentration was calculated by a triple measurement of the absorption.

5.3.2. Blank correction

Since the synthesized branched alkylthiols were not obtained as the pure desired product, and contained some amounts of the corresponding alcohols, we first had to rule out the possibility of a reaction occurring between the alcohol and the NCs surface. Blank experiments, where the branched alcohol (30 mM) was titrated into NCs solution (5.4 mM of Cd surface sites), exhibit small heat flow values of ~3μJ/s for a single injection (Figure S6a, demonstrated for 4-methyl-pentanol, 4-MPOH), which is negligible when compared to the values measured for the titration of the corresponding alkylthiols 4-MPT to the NCs (up to 30 uJ/s in absolute value. Figure S6b). The fact that the measured heat flow remains constant along the entire blank titration indicates that this is actually the mixing heat of the cell components rather than a heat change originating from a reaction of the alcohol with the NCs' surface.

For products containing ethanol residues, an endothermic feature was observed towards the end of the titration (Figure S6b). A blank titration (using the same ligand concentrations as in the ligand exchange titration) of the ethanol containing synthesized alkylthiols to TCE was found to be endothermic, with a maximum heat flow of ~10 μJ/s per injection (Figure S6c). The corrected titration curve of 4-methyl-1-pentanethiol to NCs (after blank subtraction) is presented in Figure S6d.

As an additional validation for the negligible effect of the synthesis impurities on the thermodynamics of the exchange reaction, we compared between the titration of the purchased pure alkylthiol ligand to the NC, and a solution of the ligand including the relevant impurities to the NC. We chose to demonstrate this effect on 1-octanethiol (OT). Two solutions were prepared: (1) a solution of OT (23%) and 1-octanol (77%) and (2) a solution of OT (96%) and ethanol (4%). The ratio between the ligand and the added impurities were chosen in correlation with the ratios of the products in the ligand synthesis (Table S1). As observed from the ITC data, the thermodynamics of the exchange reaction with the pure ligand and also with the non-pure solution was similar (Figure S7 and Table S4).

`

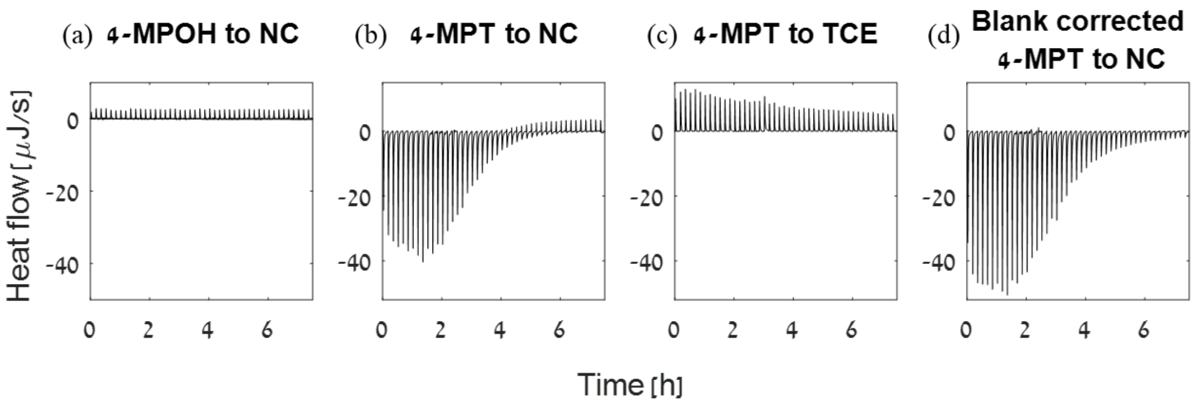

Figure S6. *Real-time thermograms for the titration of: (a) 4-methyl-1-pentanol (4-MPOH) into TCE, (b) 4-MPT (containing 4% ethanol) into oleate coated CdSe NCs, (c) 4-MPT (containing 4% ethanol) into TCE, and (d) the corrected heat flow for the titration of 4-MPT to oleate coated CdSe NCs. All experiments were performed in TCE, at 323K, with ligand concentration of 38 μM.*

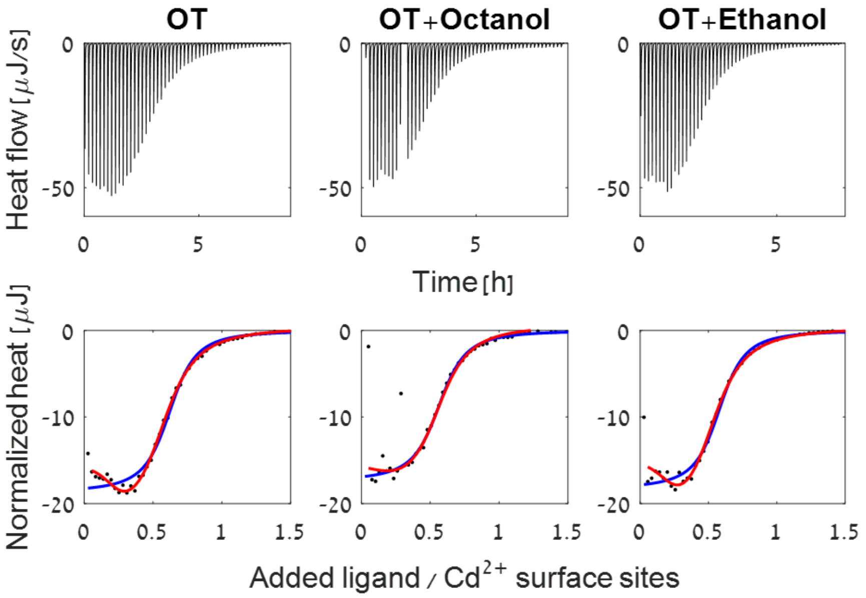

Figure S7. *Real-time thermograms and the corresponding titration curves with their model fitting for the ligand exchange reaction of oleate coated CdSe NCs with OT (pure and as non-pure solution) at 323K.*

`

Ligand	Ligand [mM]	Surface sites [mM]	Model type	ΔH [kJ/mol]	K_{eq}	n	ΔS [J/molK]	ΔG [kJ/mol]
**OT**	30	5.5	Single	-21.1	40	0.64	-35	-9.9
			Two	**-28.5**	**16**	**0.35**	**-65**	**-7.5**
				-18	201	0.21	-11	-14.2
**23% OT 77% Octanol**	30	5.8	Single	-20.8	32	0.60	-35	-9.3
			Two	**-28.0**	**19**	**0.32**	**-62**	**-7.9**
				-17	60	0.22	-17	-11.0
**96% OT 4% Ethanol**	30	5.3	Single	-20.0	36	0.60	-32	-9.6
			Two	**-27.8**	**13**	**0.32**	**-65**	**-7.0**
				-16	125	0.21	-10	-13.0

Table S4. *The thermodynamic parameters extracted from the model fit of the titration curves for the ligand exchange reaction of oleate coated CdSe NCs with 1-octanethoil at 323K, presented in* Figure S7. *The presented errors were calculated from the goodness of fitting.*

`

5.3.3. ITC data and analysis for ligand exchange with linear alkylthiols

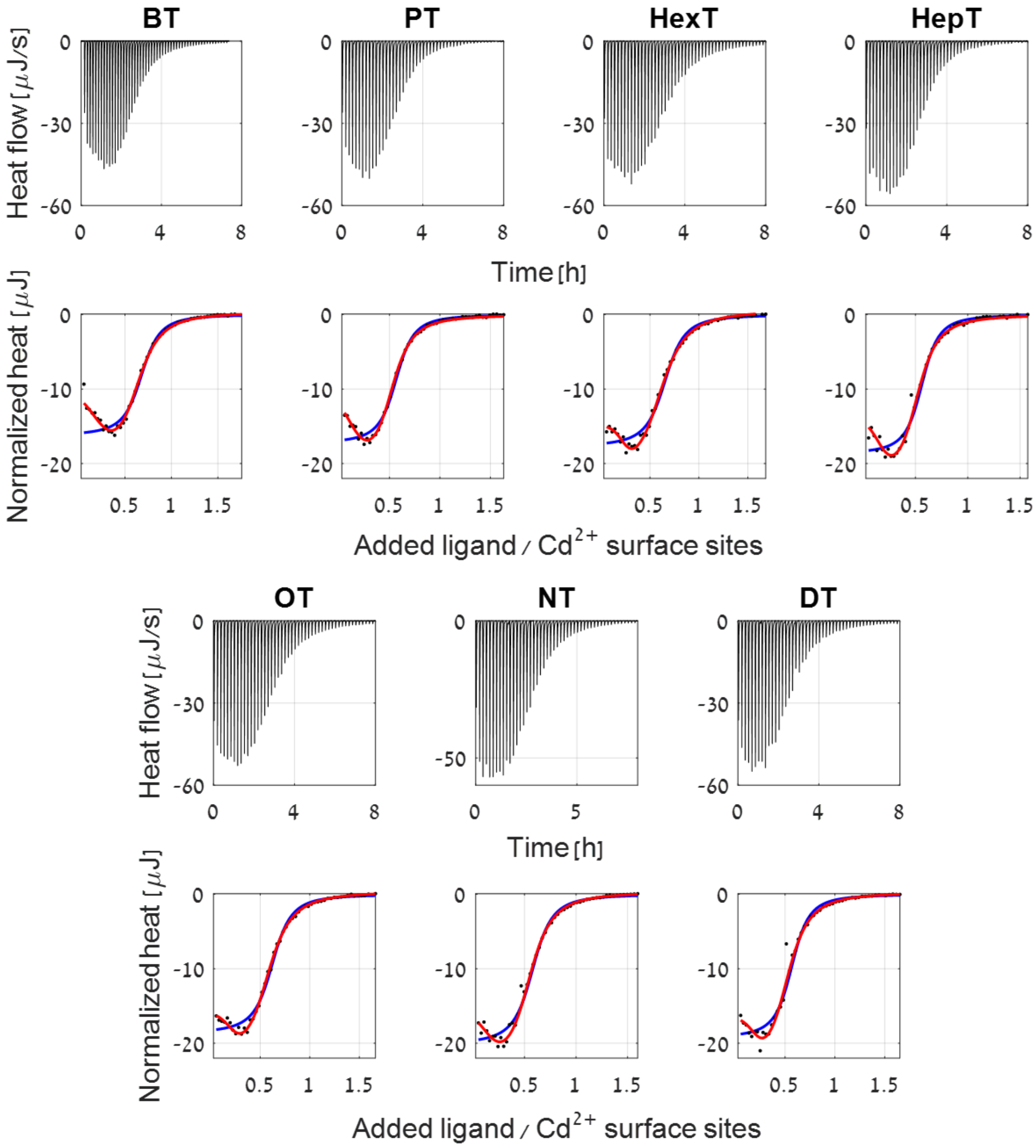

Figure S8. *Real-time thermograms and the corresponding titration curves with their single-site (blue) and two-site (red) model fittings for the ligand exchange reaction of oleate capped CdSe NCs with linear alkylthiols at 323K.*

`

Ligand	Ligand [mM]	Surface sites [mM]	Model Type	ΔH [kJ/mol]	K_{eq}	n	ΔS [J/molK]	ΔG [kJ/mol]
**1-Butanethiol (BT)**	31	5.3	Single	-18.0	44	0.71	-24	-10.2
			Two	**-26.0**	**19**	**0.38**	**-56**	**-7.9**
				-10	122	0.23	9	-12.9
**1-Pentanethiol (PT)**	30	5.7	Single	-20.0	39	0.58	-32	-9.9
			Two	**-27.5**	**21**	**0.34**	**-60**	**-8.1**
				-10	122	0.16	9	-12.9
**1-Hexanethiol (HexT)**	30	5.4	Single	-19.8	40	0.67	-31	-9.9
			Two	**-27.0**	**15**	**0.38**	**-61**	**-7.3**
				-17	307	0.22	-5	-15.4
**1-Heptanethiol (HepT)**	30	5.8	Single	-22.4	45	0.57	-38	-10.2
			Two	**-30.0**	**20**	**0.33**	**-68**	**-8.0**
				-16	198	0.17	-4	-14.2
**1-Octanethiol (OT)**	30	5.5	Single	-21.1	40	0.64	-35	-9.9
			Two	**-28.5**	**16**	**0.35**	**-65**	**-7.5**
				-18	201	0.21	-11	-14.2
**1-Nonanethiol (NT)**	30	5.6	Single	-23.4	35	0.6	-43	-9.5
			Two	**-31.2**	**19**	**0.36**	**-72**	**-7.9**
				-17	132	0.17	-12	-13.1
**1-Decanethiol (DT)**	30	5.5	Single	-22.0	40	0.58	-37	-9.9
			Two	**-29.9**	**15**	**0.31**	**-70**	**-7.3**
				-19	182	0.2	-14	-13.9

Table S5. *A table summarizing the thermodynamic parameters extracted from the single-site and two-site model fit of the titration curves for the ligand exchange reaction of oleate coated CdSe NCs with linear alkylthiol at 323K, presented in* Figure S8.

5.3.4. <u>ITC data and analysis for ligand exchange with branched alkylthiols</u>

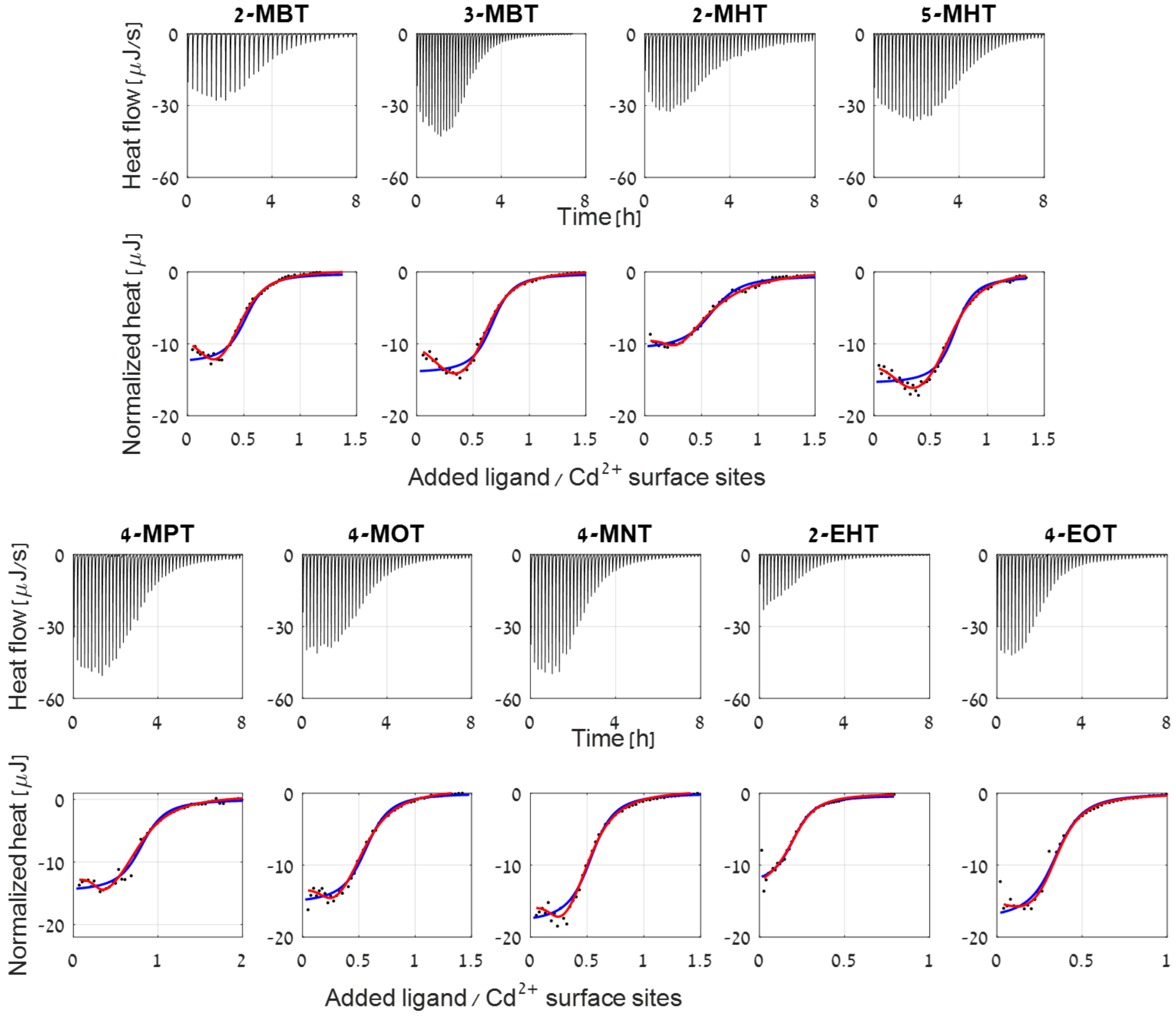

Figure S9. *Real-time thermograms and the corresponding titration curves with their single-site (blue) and two-site (red) model fittings for the ligand exchange reaction of oleate capped CdSe NCs with branched alkylthiols at 323K.*

Ligand	Ligand [mM]	Surface sites [mM]	Model type	ΔH [kJ/mol]	K_{eq}	N	ΔS [J/molK]	ΔG [kJ/mol]
**2-Methyl-1-butanethiol (2-MBT)**	25	5.5	Single	-14.1	30	0.54	-15	-9.2
			Two	**-21.0**	**11**	**0.29**	**-45**	**-6.5**
				-10	85	0.17	6	-11.9
**3-Methyl-1-butanethiol (3-MBT)**	31	5.4	Single	-15.7	51	0.68	-16	-10.5
			Two	**-24.0**	**17**	**0.35**	**-50**	**-7.9**
				-10	122	0.23	9	-12.9
**2-Methyl-1-hexanethiol (2-MHT)**	31	6.3	Single	-13.0	21	0.63	-15	-8.2
			Two	**-22.5**	**7**	**0.38**	**-54**	**-5.2**
				-9	28	0.23	-0.1	-9.0
**5-Methyl-1-hexanethiol (5-MHT)**	24	5.5	Single	-16.7	63	0.73	-17	-11.1
			Two	**-25.9**	**17**	**0.38**	**-57**	**-7.7**
				-14	175	0.24	-0.4	-13.9
**4-Methyl-1-pentanethiol (4-MPT)**	38	5.6	Single	-16.8	32	0.84	-23	-9.3
			Two	**-22.0**	**10**	**0.51**	**-49**	**-6.1**
				-15.8	457	0.26	2	-16.4
**4-Methyl-1-octanethiol (4-MOT)**	28	5.8	Single	-17.9	28	0.59	-28	-8.9
			Two	**-23.9**	**10**	**0.34**	**-55**	**-6.0**
				-16.5	174	0.2	-8	-13.9
**4-Methyl-1-nonanethiol (4-MNT)**	30	5.7	Single	-21.2	26	0.56	-39	-8.7
			Two	**-27.5**	**11**	**0.31**	**-65**	**-6.3**
				-19.5	227	0.2	-15	-14.6
**2-Ethyl-1-hexanethiol (2-EHT)**	17	6.5	Single	-15.5	11	0.23	-28	-6.5
			Two	**-16.5**	**11**	**0.23**	**-31**	**-6.5**
				-	-	0	-	-
**4-Ethyl-1-octanethiol (4-EOT)**	23	6.3	Single	-22.3	17	0.37	-45	-7.9
			Two	**-27.8**	**10**	**0.20**	**-67**	**-6.2**
				-19	107	0.14	-20	-12.5

Table S6. *A table summarizing the thermodynamic parameters extracted from the single-site and two-site model fit of the titration curves for the ligand exchange reaction of oleate coated CdSe NCs with branched alkylthiols at 323K, presented in Figure S9.*

`

6. Additional surface characterization: FTIR and TGA

Though ITC was the main method used for studying the ligand exchange reaction, we combined additional characterization techniques, discussed in details also in our previous study,[1] specifically Fourier transform infrared spectroscopy (FTIR) and thermogravimetric analysis (TGA), for determining the NCs surface composition at different stages of the ligand exchange process.

ATR-FTIR measurements were used to identify the organic species present in the NCs solution. All measured FTIR spectra for the pure ligands and the dried samples of NC are presented in Figure S10a. The spectrum recorded for the purified oleate coated CdSe NCs prior to the ligand exchange reaction (green), exhibits a sharp peak at 1540 cm^{-1}. This is assigned to the C=O group stretching mode of the oleate ligand, where the red shift from the peak position of free C=O (1710 cm^{-1}, as seen in the spectrum of free oleic acid, Figure S10a, purple) is a signature of surface bound oleate.[9] This peak vanishes completely upon ligand exchange with 4-MNT under non-ITC conditions (excess of 5 ligands per Cd surface site, cyan), indicating the exchange is full under these conditions. The spectrum of the crude ITC exchanged NCs (blue), features both states; a significant peak at 1710 cm^{-1} indicates the release of considerable amount of oleate ligands due to their exchange with 4-MNT ligands. In addition, we observe the peak at 1540 cm^{-1}, which is assigned to a portion of remaining bound oleate ligands. The appearance of both peaks in this spectrum suggests that the ligand exchange performed under ITC conditions is incomplete, with some amount of unexchanged surface sites. After purification of the crude ITC solution (purified, red), the peak representing the free oleate ligands disappears, since all non-surface bound ligands are discarded during the purification process.

Further analysis of the ligand layer composition was obtained by TGA, where the differences in the overall mass loss and in the shape of the TGA thermograms upon ligand exchange indicate the changes in the surface ligand layer composition. TGA results for the samples discussed above are presented in Figure S10b. First, we performed this analysis for the oleate coated NCs (green thermogram), in order to determine their initial surface coverage. The total mass loss up to 500°C, which is attributed to any organic species present in the sample, was 44%, consistent with a full surface coverage and a 1:1 binding ratio (all Cd surface sites are bound to a single oleate ligand). The mass loss of the 4-MNT coated, non-ITC exchanged NCs (cyan thermogram), was 30%, which also correlates with a full surface coverage and a 1:1 ratio of thiols to Cd surface sites, confirming the complete ligand

`

exchange under these experimental conditions. In TGA thermograms, the temperatures of the inflection points are used to identify the surface composition. The thermogram of oleate coated CdSe NCs (green) exhibits a single inflection point at 340°C, indicating the expected presence of oleate ligands only, while the thermogram of 4-MNT coated NCs, achieved by ligand exchange under non-ITC conditions, exhibits a single inflection point at 280°C (cyan arrow), indicating a complete ligand exchange has occurred. However, following a ligand exchange under ITC conditions (purple thermogram), the thermogram exhibits multiple inflection points, evidencing a mixture of ligands on the NCs surface as a result of incomplete exchange. This is further supported by the amount of organic content in the ITC exchanged sample; as mentioned above, 3nm CdSe NCs with their surface fully covered with only 4-MNT ligand should contain 30% of organic content. But in the ITC exchanged sample the organic content was higher, with a measured mass loss of 34%, consistent with the higher molecular mass of oleate ligands compared to that of 4-MNT. After a partial exchange, some of the NC surface sites are still bound to the heavier oleate ligands, raising the percentage of organic content in the TGA sample. Those observations are also consistent with the FTIR results, discussed above, which indicated the presence of bound oleate upon ligand exchange in ITC conditions, while not present under non-ITC conditions.

We believe the observed difference in the extent of the exchange reactions stems from the variation in the experimental conditions. Under non-ITC conditions, the system equilibrium is immediately disturbed by the quick addition of a large excess of the exchanging ligand (5:1 alkylthiols to Cd surface sites). In contrast, during the ITC measurement a small amount of alkylthiol is added to the NCs, and the system returns to equilibrium after each step. The final excess of alkylthiol is only 1.5-2, a ratio which is insufficient to induce complete ligand exchange. The performance of ITC measurement in the described non-ITC conditions would not allow the extraction of the reaction thermodynamics parameters since an informative titration curve would not be attained.

In order to estimate the ligands composition for the post-ITC NC samples, we divided the TGA thermograms into 2 regions: 110-315°C, which is mainly attributed to bound alkylthiolate (Figure S10b, red region), and 315-500°C, which is mainly attributed to bound oleate (Figure S10b, green region). The chosen regions are correlated with the temperature inflection points of the ligands. In addition, we verified that the residues of the mass loss beyond the mentioned regions are negligible for the fully coated NCs with either oleate (6%) or 4-MNT (non-ITC, 10%). The TGA thermogram and the ligand composition results for the linear and branched ligands discussed in the main text are presented in Figure S11 and Table

S7, respectively. We note that all TGA analysis can have up to 10% of inaccuracy due to the different purification efficiency of each sample.

Generally, the coverage of the linear alkylthiolate is higher than the branched ligands. Moreover, the coverage of the branched ligand with branching group located closer to the NC surface is even lower. Those observations are in correlation with our conclusions regarding the steric hindrance in branched ligand packing on the NC surface, which also affects the Gibbs free energy values for each of the ligand exchange reactions, as discussed in the main text.

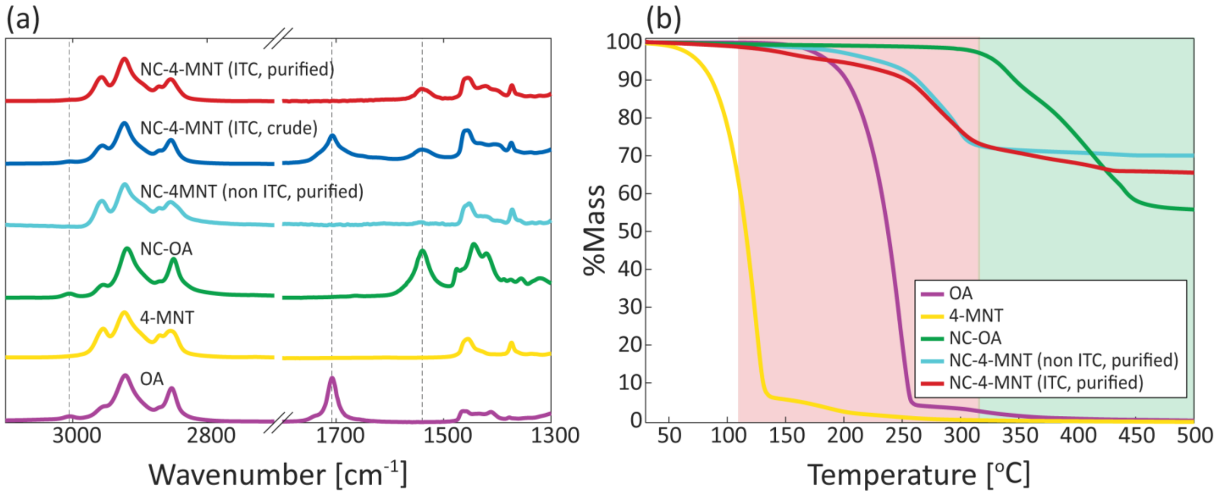

Figure S10. *(a) FTIR spectra of CdSe NCs before (purified, green) and after ligand exchange reaction with 4-MNT under ITC conditions (crude−blue, purified−red), and under non-ITC conditions (cyan), and the pure oleic acid (OA, purple) and 4-MNT (yellow) ligands. (b) TGA thermograms of purified CdSe NCs before (green) and after ligand exchange with 4-MNT under ITC (red) and non-ITC (cyan) conditions. The thermograms of the pure ligands (OA−purple, 4-MNT−yellow) are also presented. The differences in both the temperature of inflection points and the loss of organic mass, indicate the changes in the surface ligands.*

We also observed a general trend of lower alkylthiolate coverage for shorter ligands. We assume the source for this trend is actually an error in the chosen section, as the inflection point for shorter ligands is higher than for the longer one (Figure S11a), which was kept constant to maintain consistency throughout the analysis. Although this is conflicting with the inflection points trend for the free alkylthiols, in the case of bound ligands there is also an effect of the binding strength. We assume that the higher polarity of the shorter ligands results in stronger NC surface-ligand bond which requires higher temperature in the TGA to detach the ligand. Therefore, we used only the average of the TGA results to justify the ligand coverage in the simulation discussed ahead.

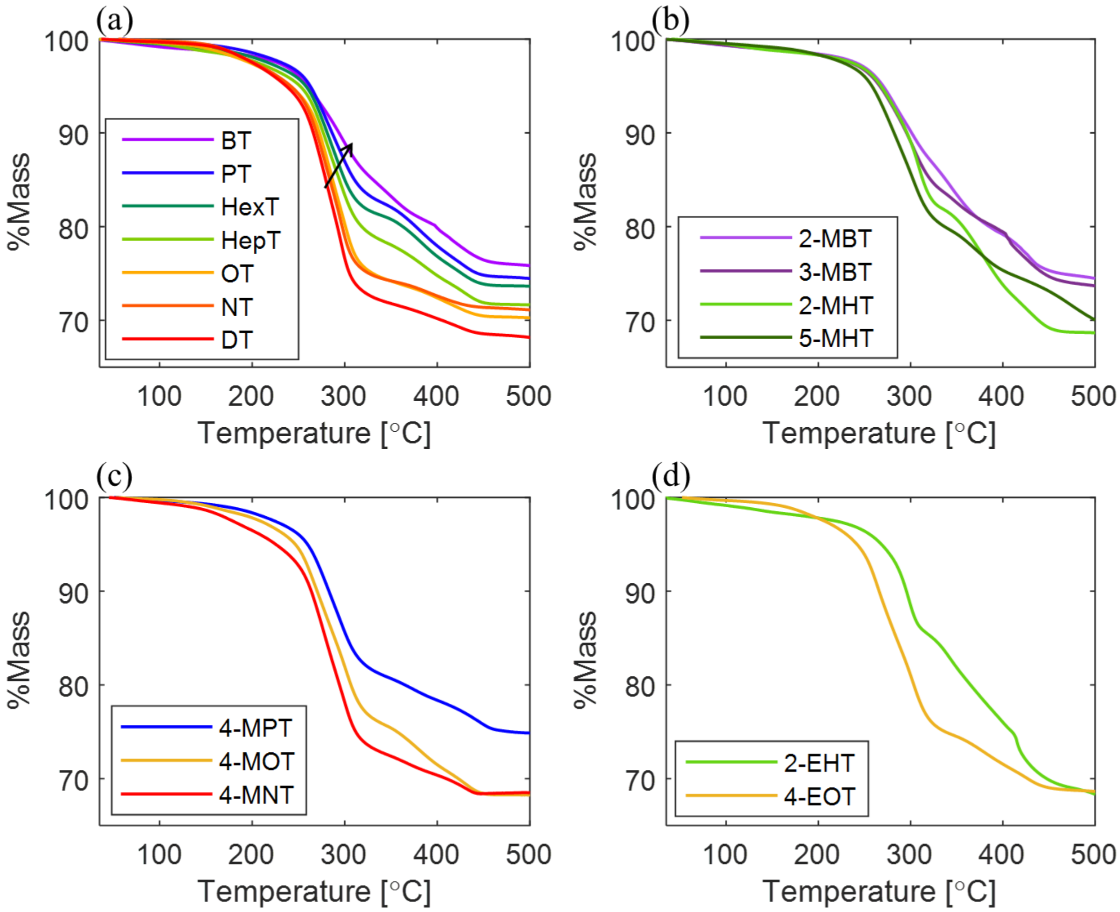

Figure S11. *TGA thermograms of purified CdSe NCs after ITC-performed ligand exchange reactions with (a) linear (arrow indicates the shift in the inflection point with changing ligand length) and (b-d) branched alkylthiols.*

`

Surface ligand	%Thiolate (110-315 °C)	%Oleate (315-500 °C)	Total coverage
**1-Butanethiol (BT)**	71%	20%	91%
**1-Pentanethiol (PT)**	77%	18%	95%
**1-Hexanethiol (HexT)**	75%	17%	92%
**1-Heptanethiol (HepT)**	76%	19%	95%
**1-Octanethiol (OT)**	88%	18%	100%
**1-Nonanethiol (NT)**	82%	9%	91%
**1-Decanethiol (DT)**	86%	10%	96%
**2-Methyl-1-butanethiol (2-MBT)**	57%	24%	81%
**3-Methyl-1-butanethiol (3-MBT)**	67%	23%	90%
**2-Methyl-1-hexanethiol (2-MHT)**	64%	31%	95%
**5-Methyl-1-hexanethiol (5-MHT)**	72%	24%	96%
**4-Methyl-1-pentanethiol (4-MPT)**	74%	19%	93%
**4-Methyl-1-octanethiol (4-MOT)**	77%	20%	97%
**4-Methyl-1-nonanethiol (4-MNT)**	82%	12%	94%
**2-Ethyl-1-hexanethiol (2-EHT)**	52%	35%	87%
**4-Ethyl-1-octanethiol(4-EOT)**	73%	17%	90%

Table S7. *Post-ITC ligand composition, as calculated from the TGA results presented in Figure S11.*

`

7. Conformational entropy calculation

7.1. Conformational entropy of free ligands

The conformational entropy of ligands can be calculated by enumerating ligand conformations. The conformations of the free ligand were determined as following.[10] For a linear alkylthiol, with the general structure $CH_3(CH_2)_{n-1}$-SH, all n bonds (including S-C bond) are considered to have three possible conformations, (denoted by c): t, g^+, g^- (corresponding to dihedral angles of 0°, 120° and -120°, respectively). An energy difference of 500 cal/mol between t and g^+ or g^- states was considered. Conformations forbidden due to steric exclusion between non-neighboring carbons at a distance shorter than the allowed van der Waals radius for CH_2 group were eliminated. Furthermore, conformations with sequence g^+g^- or g^-g^+ between neighboring bonds (termed as second order interaction) were excluded due to their high energy. As expected, the total number of conformations increases exponentially with ligand length (Figure S12a).

In addition to the conformational degrees of freedom, for every conformation c the chain is allowed to freely rotate in the space, resulting in energetically degenerate orientations (denoted by o). All allowed sampled states of the chain, (denoted $s=\{c,o\}$) were included in the conformational entropy calculation of the free chain, within the usual expression for the canonical ensemble entropy. The corresponding partition function is

(S31) $$Z = \sum_i g_i \exp\left(-\frac{E_i}{RT}\right)$$

where E_i is the conformational energy of state i per mole (derived from the sum over all bonds, considering energy value of 0 for t and 500 cal/mol for g^+ and g^-), T is taken as the experimental temperature of 323 K, R is the gas constant, and g_i is the degeneracy of the state arising from the free rotation of the chain in the space (which is equal for all conformations). The mean conformational energy is given by

(S32) $$\langle E_{conf}\rangle = \frac{1}{Z}\sum_i E_i g_i \exp\left(-\frac{E_i}{RT}\right)$$

with corresponding conformational entropy

(S33) $$S = R\ln Z + \frac{\langle E_{conf}\rangle}{T}$$

As expected, both the mean conformational energy and the conformational entropy increase with the ligand length (Figure S12b and S12c, respectively). The elimination of high energy (low probability) conformations with sequences g^+g^- or g^-g^+ did not affect the values of the mean energy and entropy.

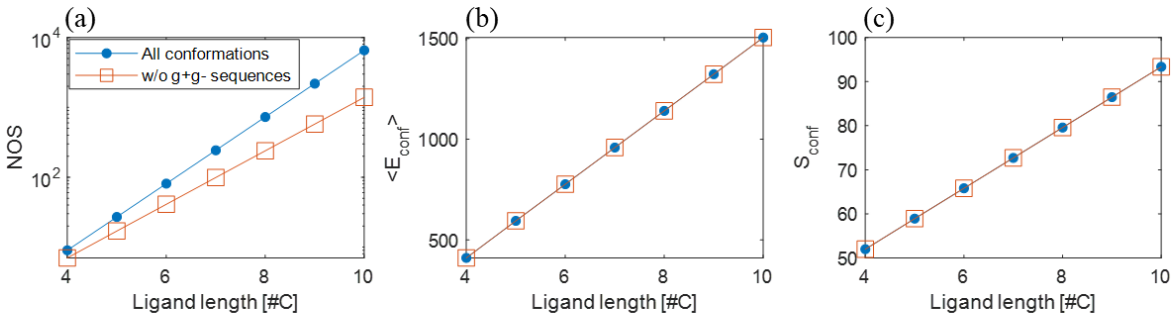

Figure S12. *Calculation results for free linear alkylthiol with 4 to 10 carbons. (a) Total number of available conformations (blue dots), and the number after excluding the high energy* g^+g^- *or* g^-g^+ *sequences (red squares). (b) Calculated mean conformational energy and (c) conformational entropy with (blue dots) and without (red squares) the high energy sequences.*

7.2. Conformational entropy of NC-bound ligands

As was mentioned in the main text, mean-field theory, previously presented for micellar systems, membranes and grafted polymer brushes, was applied to calculate the conformational entropy of NC-bound ligands. The theory enables determining the probability distribution function (PDF) of the allowed conformations of the ligands subjected to the packing constraints of chains attached to the NC. To simulate experimental conditions, all calculations were performed for a ligand bound to a sphere with diameter of 3 nm.

7.2.1. Calculations for frozen and free anchored ligands:

Using all calculated states of the free ligand, the allowed accessible states of the free anchored ligand were derived by eliminating states where the ligand overlaps and penetrates into the NC surface. Considering the probabilities, P_s, of each accessible ligand state, the conformational Gibbs entropy is

(S34) $S = -R \sum_s P_s \ln P_s$

As detailed in the main text, the conformational entropy of the frozen ligand was considered to be zero. Figure S13 shows the entropy change upon ligand binding for free anchored (blue circles) and frozen ligands (purple triangles). The experimental results (black squares) lie between the two extreme calculated conformational entropy changes for the fully frozen and free anchored ligands.

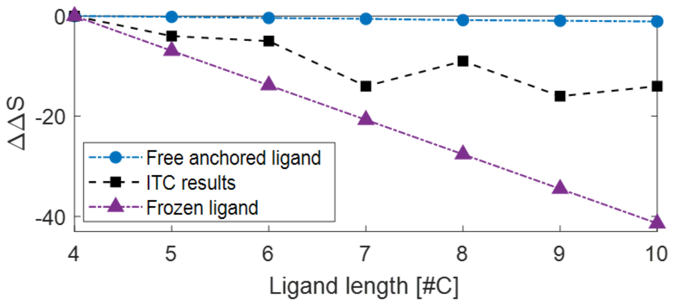

Figure S13. *ITC experimental data (black squares) and calculated conformation entropy for free anchored (blue circles) and frozen (purple triangles) ligands. Data are presented as the difference in the entropy from the shortest BT ligand.*

7.2.2. Entropy of constrained ligands

The free energy of ligands on the surface is minimized subject to packing constraints. The Helmholtz free energy for a chain (per mole) is

$$(S35) \quad A = E - TS = \sum_s P_s \epsilon_s - TR \sum_s P_s \ln P_s$$

where P_s and ϵ_s are the probability and conformational energy for state s, respectively, T is the experimental temperature (323 K) and R is the gas constant. For chains in the bad solvent regime the probabilities, P_s, are subject to the liquid close-packing or hydrocarbon liquid density constraints of the ligand shell volume. This can be expressed in terms of a Lagrange multiplier coupled to the constant density constraint, resulting in the following single-chain conformational probability[11]

$$(S36) \quad P_s = \frac{1}{Z} \exp\left(-\beta\epsilon_s - \beta \sum_i \pi_i \cdot v_{CH_2} \cdot N_{CH_2,i}\right)$$

where Z is the partition function, π_i is the Lagrange multiplier representing lateral pressure in layer i within the ligand shell, v_{CH2} is the average volume of a CH_2 group in the bulk liquid state, and $N_{CH_2,i}$ is the number of CH_2 groups located within layer i. Since $v_{CH_3} \cong 2v_{CH_2}$ the terminal CH_3 group counts as two segments.

By dividing the ligand shell into L layers (Figure 5a in the main text), we calculated the number of -CH_2 segments, N_{CH_2}, residing within each layer for every accessible s state.

Consider first chains that are constrained to densely pack close to the NC surface.[12] A chain with the structure $CH_3(CH_2)_{n-1}$- has a volume of $(n+1) \cdot v_{CH_2}$. Hence, the profile density of the bound ligand shell starts with a constant maximal density, $\rho_0 = 1/v_{CH_2}$, up to the last layer j for which $\rho_0 \mathrm{v_j}$ gives higher number of segments than there are in the chain, resulting in a layer density that is lower fit the packing requirement,

$$(S37) \quad \sum_i \langle N_{CH_2} \rangle_i = \sum_{i=1}^{L} \rho_i \mathrm{v_i} = n + 1$$

In this expression, the sum is taken over all L layers, $\langle N_{CH_2}\rangle$ is the average number of CH_2 segments, ρ_i is segments density, and $v_i = N_{ligand}/V_i$ is the volume available per ligand in the layer, calculated from the total number of ligands, N_{ligand} (determined from TGA measurements, as discussed in the previous section), and layer volume, V_i. The segment density in layers with $i>j$ is $\rho_i = 0$ (Figure S14a, for representative short PT and long DT ligands). This density profile is then used to find the ligand shell profile by substituting eq. (S36) to eq. (S37), considering $\langle N_{CH2}\rangle = \sum N_{CH2} \cdot P_s$, so that

$$\text{(S38)} \quad \sum_s [N_{CH2,i}(s) - \rho_i v_i] \exp\left(-\beta\epsilon_s - \beta \sum_i \pi_i \cdot v_{CH2} \cdot N_{CH2,i}\right) = 0 \qquad i = 1, \ldots L.$$

The lateral pressures for all layers, π_i , are determined by the numerical solution of the set of L equations. The calculated P_s fully recover the required density profile of eq. (S37) (Figure S14b). As expected, the lateral pressure decreases with increasing distance from the NC surface, because the layer is less occupied by segments, and are thus less affected by the packing constraints (Figure S14c).

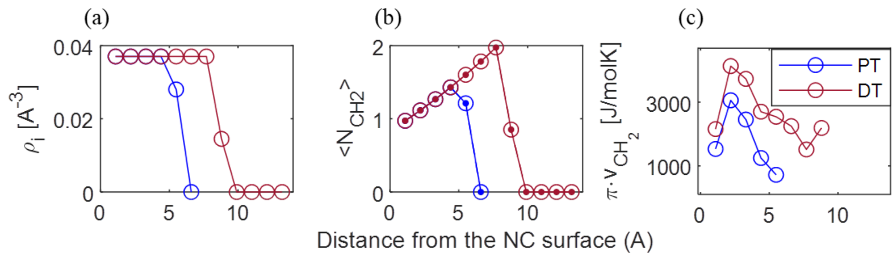

Figure S14. *Calculation results for bound linear alkylthiol with 5 (blue) and 10 (red) carbons. (a) Density profile of the ligand shell. (b) Average number of CH_2 segments at each layer. The calculated probabilities (circles) fully recover the required density profile (dots). (c) The calculated lateral pressure for the occupied layers.*

The presented packing constraint, which determines a constant bulk-liquid density for the ligand shell, is correlated with the "bad solvent" regime, as the ligand chains alone occupied the entire volume near the NC surface. In order to corroborate how appropriate is in this regime for the bound ligands in TCE, we examine the other extreme case of the "good solvent" regime, whereby the solvent molecule freely penetrate the ligand shell and mix and pack together with the ligands. The calculation rout is similar to the one presented for the "bad solvent" regime, but the packing constraint is altered to include the solvent molecules volume. The entropy of such a system is composed of two contributions: the ligand chain and the solvent molecules, as follows[13]

`

(S39) $S = -R\sum_s P_s \ln P_s - R\sum_i \rho_{solvent} \ln[\rho_{solvent} \cdot \nu_{solvent}]$

where P_s is the probability for state s (defined in eq. (S36)), and ρ_{solvent} and ν_{solvent} are the density profile of the solvent and the volume of a solvent molecule, respectively,

(S40) $\rho_{solvent} = \frac{1}{\nu_{solvent}} \exp(-\beta\pi_i v_{solvent})$

Here, π_i is the lateral pressure in layer *i* within the ligand shell. In all our calculations we used the parameters from TCE to simulate the environment of the ligands in the experiments.

The lateral pressure at each layer minimizes the free energy subject to the requirement that each layer volume (v_i) is entirely filled by either chain segments or solvent molecules:

(S41) $v_i = \sum_s [v_{CH_2} \cdot N_{CH2,i}(s) \cdot P(s)] + [\rho_{solvent} \cdot \nu_{solvent} \cdot v_i] \qquad i = 1, \ldots L$

Figure S15 shows calculation results for representative short and long ligands- 1-pentanethio (PT) and 1-decanethio (DT). The calculated P_s fully recover the input of eq. (S41) (Figure S15a). As expected, the lateral pressure decreases with increasing distance from the NC surface, because the layer is less occupied by ligand chains (Figure S15b). The lateral pressure for the “good solvent” regime is slightly lower than the one calculated for the “bad solvent regime”, because the packing constraints are more relaxed as the layer is occupied also by solvent molecules. This is corroborated by the lower average number of CH_2 segments that are in close proximity to the NC surface (Figure S15c) and their density (Figure S15d). Consequently, the ligand is spread out farther from the NC surface, as observed from the probability distribution for the location of the terminal CH_3 group (Figure S15e) and also from the radius of gyration (Figure S15f), defined as

(S42) $R_g = \sqrt{\langle x^2 \rangle + \langle y^2 \rangle}$

where *x* and *y* are the coordinates of each of the chain segments in the plane perpendicular to the normal to the NC surface (the z axis). The average is taken over all the allowed states, based on the extracted single-chain PDF.

The chain conformational entropy, calculated from the first term in eq. (S39), shows the expected trend of increasing entropy loss with increasing ligand length. The values are of similar magnitude to the ones calculated in the “bad solvent” regime; however, no “odd-even” behavior is observed, as the solvent molecules interfere and relax the ligand-ligand (interactions that are strongest in the bad solvent regime (Figure S15g).

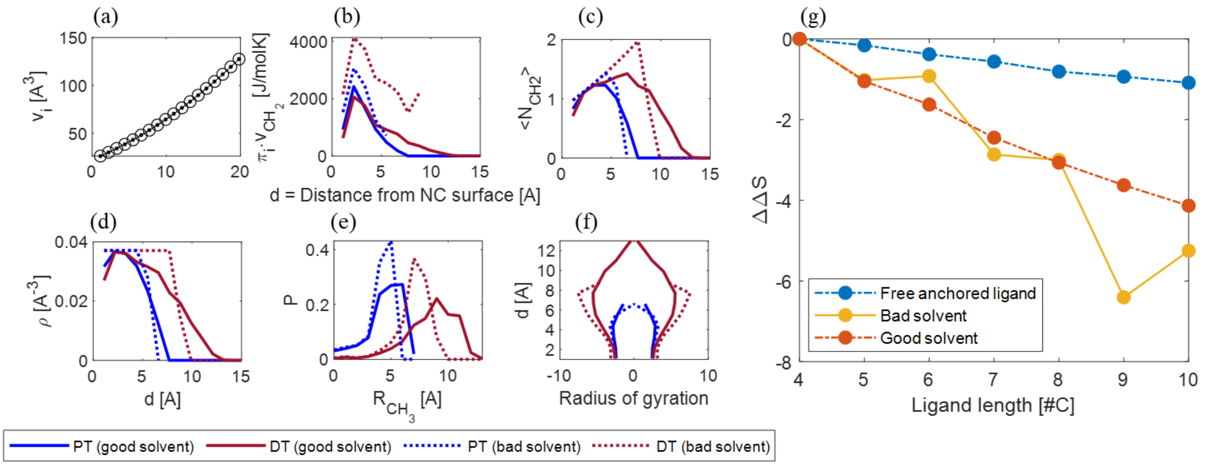

Figure S15. *Calculation results for bound linear alkylthiol with 5 (blue) and 10 (red) carbons, in the "good solvent" regime (solid line), in comparison to the "bad solvent" results (dashed line). (a) The available volume per ligand (dots) and the volume occupied by either ligand or solvent molecules according to the calculation results (circles). (b) The calculated lateral pressure. For good solvent, layers without ligands were also included in the calculation, as they still include solvent molecules. (c) Average number of CH_2 segments, calculated from the extracted probabilities. (d) CH_2 segment density. (e) Probability distribution for the distance of the terminal CH_3 group from the NC surface. (f) Bound ligand radius of gyration. (g) Conformational entropy changes upon ligand binding, for gree anchored ligand (blue), ligand in bad solvent (yellow), and ligand in good solvent (orange).*

7.2.3. Odd-even effect in conformational entropy change upon ligand binding

To find the source of the odd-even effect in the conformational entropy, we analyzed several parameters related to the packing of the ligands on the NC surface. First, we examined the change in the order parameter of the terminal bond, namely the average angle between the CH_2-CH_3 bond in the linear alkylthiol and the normal to NC surface. This parameter has previously been correlated with odd-even effects in self-assembled monolayers.[14,15] Figure S16 presents the results of the order parameter for a free anchored ligand, a ligand in a good solvent, and a ligand in a bad solvent. As we have already demonstrated, only ligands in a bad solvent exhibit the odd-even effect in conformational entropy. Similarly to the entropy, the order parameter changes monotonically with the chain length for the free anchored ligand. For a ligand in a bad solvent, we find a non-monotonic change with the odd-even behavior in both the entropy and the order parameter. By contrast, for a ligand in a good solvent we find an initial non-monotonic change in the order parameters (up to 8 carbons), while no odd-even effect was observed in the entropy. Overall, the change in the order parameter is moderated in

the good solvent regime (sum of absolute differences between odd and even chains between 4 and 8 carbons is 0.15) and is similar to the changes observed for free anchored ligand (0.11), while the change in the “bad solvent” regime is higher (0.23). The odd-even behavior is sharper for the low coverage NC, both in the entropy and the order parameter (0.29). Thus, we conclude that the order parameter of the terminal bond indeed plays a part in the odd-even effect of the conformational entropy, yet, we assume there are additional factors which have not been considered hitherto.

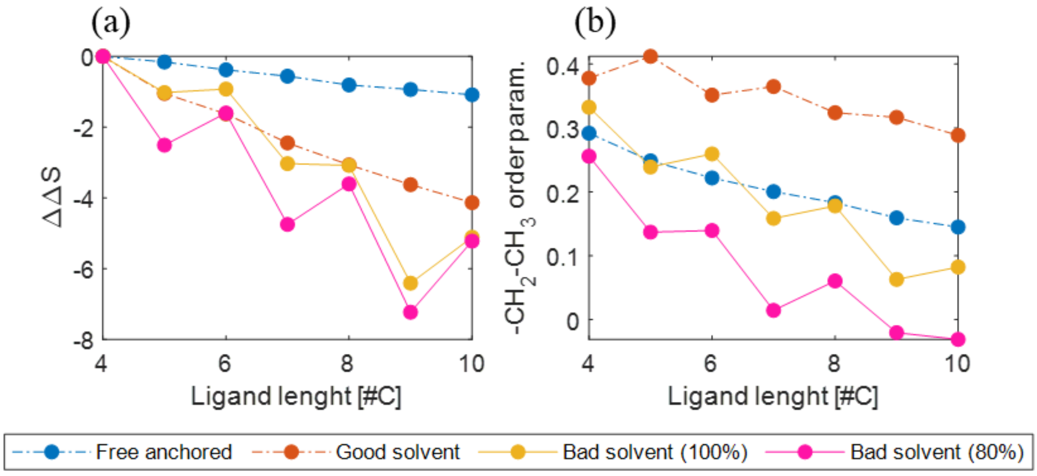

Figure S16. *(a) Conformational entropy and (b) order parameter of the terminal bond for surface bound alkylthiol for lengths of 4 to 10 carbons under different binding conditions.*

7.2.4. *Activity coefficient according to Flory-Huggins theory*

In order to find the contribution from non-ideal mixing to the overall entropy change, we used the Flory-Huggins entropy of mixing.[16–18] Because the calculation describes the changes the alkylthiol ligand undergoes (and not the oleic acid), we refer to the chemical reaction of ligand binding (NC refers to a surface site):

$$(S43)\quad NC + R - SH \rightleftharpoons NC - SR$$

At equilibrium, the chemical potentials of the components on both sides of the chemical equation are equal, and the standard Gibbs free energy change ΔG^o is given by

$$(S44)\quad \Delta G^o = -RT\ln\left[\frac{a_{NC}}{a_{RSH}\cdot a_{NC-SR}}\right] = -RT\ln\left[\frac{C_{NC}}{C_{RSH}\cdot C_{NC-SR}}\cdot\frac{\gamma_{NC}}{\gamma_{RSH}\cdot\gamma_{NC-SR}}\right]$$

Here, a is the activity, given by the component concentration C and its activity coefficient γ. In our case, ΔG^o is affected by the conformational changes of the chain upon binding, and the activity coefficient represents the non-ideality of the components in solution. Therefore, eq. (S44) can be recast as:

$$(S45)\quad \Delta G^o = -RT\ln K_c + RT ln\gamma_{RSH}$$

`

where K_C is the equilibrium constant, which is written in terms of the concentrations of the components (rather than their activities). The model used to analyze the ITC curves assumes ideal solutions as does the equilibrium constant K_C, used directly to calculate ΔG_{ITC} (eq.(S23)) and therefore also ΔS_{ITC}. Hence, the experimentally extracted ΔG_{ITC} deviates from the standard ΔG^o by the activity coefficient:

(S46) $\Delta G_{ITC} = \Delta G^o - RTln\gamma_{RSH}$

Therefore, the entropic part calculated from the reaction Gibbs free energy is

(S47) $\Delta S_{ITC} = \Delta S^o + Rln\gamma_{RSH}$

where the standard entropy change ΔS^o is the calculated change in the conformational entropy of the ligands upon binding.

The non-ideality in solution stems from the different volume of the component and the solvent molecules, as well as interactions between them. As the NC volume is affected mainly by the inorganic part, which remained unchanged, the main non-ideal contribution arises from the free ligand in the solution. While the shorter BT ligand has similar molecular volume to the TCE solvent, this is certainly not the case for longer ligands. Therefore, Flory-Huggins' (FH) theory, which is commonly applied for polymer chains, can provide an estimate for the missing activity coefficients. Within FH theory, the activity coefficient of the free alkylthiol (RSH), mixed with the solvent (TCE) can be written as

(S48) $\ln a_{RSH} = \ln \phi_{RSH} - \left(\frac{V_{RSH}}{V_{TCE}} - 1\right) \phi_{TCE} + \chi V_{RSH} \phi_{TCE}^2$

where ϕ_i and V_i represent the molar fraction and the molar volume of the components, respectively, and χ is the interaction parameter, which represents the change in the overall interactions of the system upon mixing pure alkylthiol ligands with pure TCE solvent molecules. Therefore, the FH activity coefficient is

(S49) $\ln \gamma_{RSH} = \ln V_{RSH} - \left(\frac{V_{RSH}}{V_{TCE}} - 1\right) \phi_{TCE} + \chi V_{RSH} \phi_{TCE}^2$

The Flory-Huggins' interaction component χ contributes mostly to the enthalpy (which is not treated here), but may also have an entropic contribution.

7.2.5. Flory-Huggins interaction parameter

Flory-Huggins' interaction parameter was used in this work as a fit parameter for the entropy change upon ligand binding. The addition of this parameter to our calculation is justified by the non-zero mixing enthalpy of the investigated alkylthiol and the solvent TCE. Since the mixing reaction is exothermic, it is very plausible that the solvent molecules tend to organize with the alkylthiol molecules, hence suggesting a compensating loss of entropy upon

`

mixing. Furthermore, it was already observed in many systems that this parameter is temperature dependent, and thus has a contribution to the entropy change upon mixing.[19] There are also several works that support our choice to use a chain-length dependent parameter, which decreases with increasing ligand length.[20–22] ITC dilution (mixing) experiments between pure alkylthiol titrated into pure TCE also corroborate our assumption that the interaction parameter decreases with increasing ligand length. For example, comparing the mixing enthalpy of OT and HexT reveals that the latter is bigger (Figure S17). Note that the enthalpy is calculated from regular solution theory as:

(S50) $$\Delta H_{mix} = \frac{Q}{N_{total}} = wRT \cdot \xi_{TCE} \cdot \xi_{RSH}$$

where Q is the accumulated heat as measured by ITC, N_{total} is the total number of TCE and alkylthiol (RSH) molecules in the ITC cell after each injection, w is the mixing interaction parameters, and ξ_{TCE} and ξ_{RSH} are the mole fractions of the TCE and alkylthiol molecules in the ITC cell, respectively. Therefore, the enthalpic interaction parameter (which is distinct from the FH interaction parameters for the entropy, but should behave similarly with changes in the ligand structure) is extracted from the slope of the curves presented in Figure S17f).

The correlation of FH parameter to the backbone length rather than to other previously used parameters, such as molar mass,[20,22] is corroborated by the dilution experiments, because we have found that the enthalpy of 2-EHT is closer to the one measured from HexT (similar backbone length of 6 carbons) than for OT (similar total number of 8 carbons). This also points to higher FH parameter for branched ligand, in consistent with some previous works.[23] Thus, we used the simple relation between the interaction parameter and the ligand length which is resulted in good agreement between the calculated and the experimental entropy change:

(S51) $$\chi = \frac{0.3}{Number\ of\ carbons}$$

We assume that the main exothermic feature comes from the interaction between the polar TCE molecule and the thiol polar head group. This assumption is also supported by the endothermic mixing between TCE and hexane, which is a non-polar molecule. In addition, this assumption justifies our use of the “bad solvent” regime in our conformational entropy calculation, as the solvent molecules might lose both enthalpy and entropy upon penetrating to the ligand shell, as the polar thiol attaches to the NC surface and is hardly available to interact with the solvent. On the other hand, the colloidal stability of the NC in solution points to a “good solvent” nature, and therefore we assume that the system is in an intermediate regime between the two extreme regimes.

`

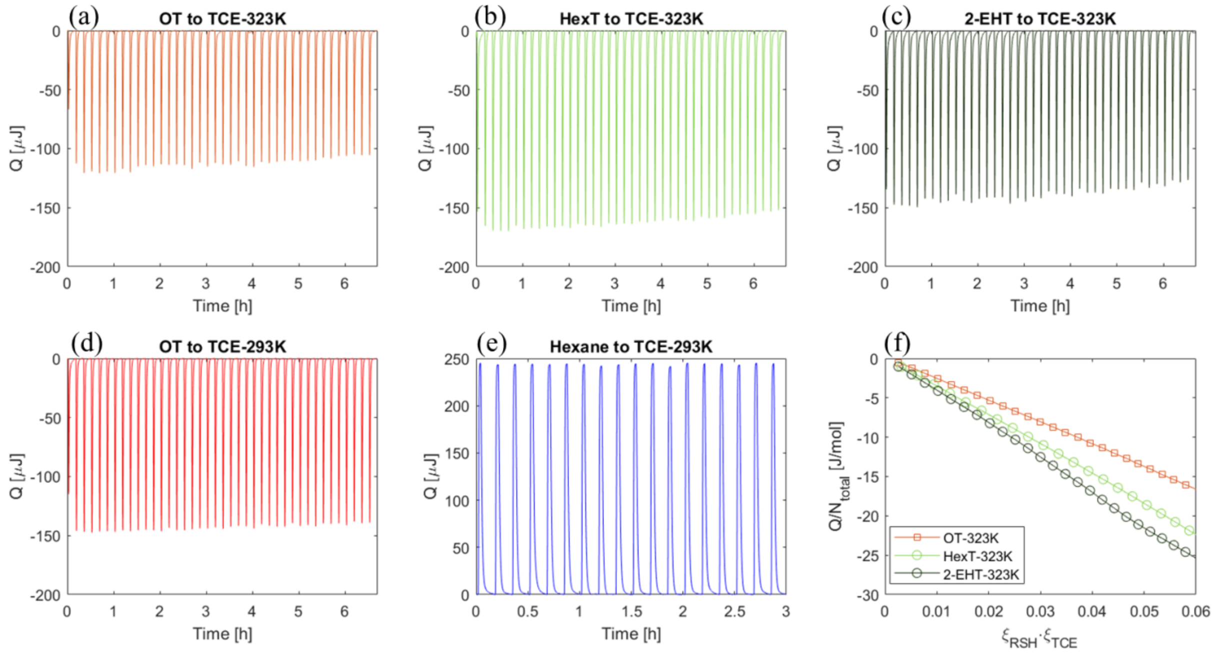

Figure S17. *Real-time thermograms of the mixing of pure alkylthiol with pure TCE: (a) OT (red square), (b) HexT (light green, circles), and (c) 2-EHT (dark green circles), at 323K, and (d) OT at 293K. (e) Real-time thermograms of the mixing of pure hexane with pure TCE at 293L. (f) The total accumulated heat, normalized to the total numbers of alkylthiols and TCE, is presented as a function of the multiplicity of the molar fractions of the alkylthiol and the TCE.*

7.2.6. Conformational entropy for branched ligands

The conformations of the branched alkylthiol ligands are derived similarly to the ones of the linear ligands. However, the energy differences between *t*, g^+, and g^- conformations involving the bonds adjacent to the branching group, as well as an energy correction derived from second order interactions with the branching group, were altered considering the proximity between the branching group and the other chain residues in each conformation.[10]

As described in the main text, in the case of chiral branched ligands all the experimental data were collected for racemic mixtures. For simplification (and for minimizing the computational effort), we presented the conformational results for the R enantiomer only. Calculating the conformational entropy of the S enantiomer resulted in similar trends, as presented in Figure S18.Chiral packing constraints were not considered in the current study.

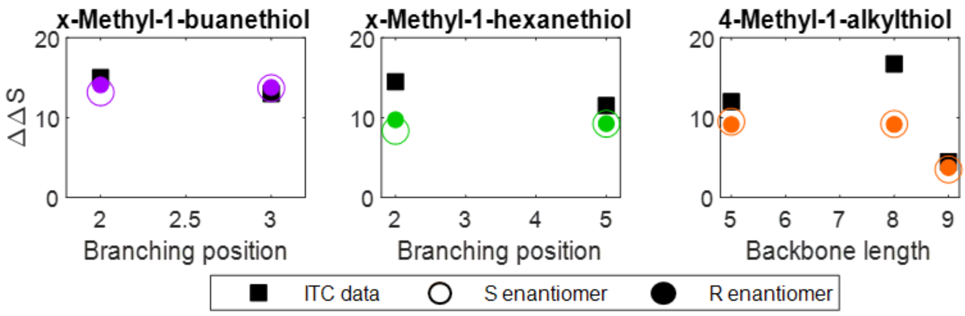

Figure S18. *ITC experimental data (squares) and conformational entropy calculation results for R- (full circle) and S- (hollow circle) methyl branching alkylthiols. All results are presented as the difference in entropy between the branched ligand and the corresponding linear ligand with the same total number of carbons.*

Although the calculated entropy that considers the experimentally observed alkylthiol coverage (Figure S11 and Table S7) reproduces most of the experimental ITC results, we also analyzed the effect of the alkylthiol coverage itself. The differences in the entropy between the non-iso branched alkylthiols and the corresponding linear ligands are smaller when using the same 80% surface coverage (Figure S19, empty circle). The difference in entropy is even smaller when assuming full coverage for both types of ligands (Figure S19, empty triangle). While there is still entropic preference for the binding of branched over linear ligands, the alkylthiol coverage indeed affects the overall entropy change. Packing branched ligands requires additional volume, especially when the branching group is located near the NC surface. Therefore, forcing their organization at higher surface density decreases their entropy.

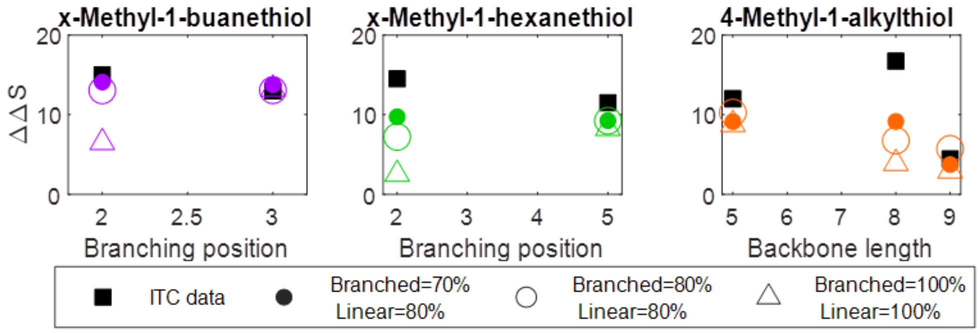

Figure S19. *Surface coverage effect on the overall entropy difference between branched and linear ligands. Total entropy change upon alkylthiol binding was calculated for 70% (full circles, presented also in Figure 6 in the main text), 80% (empty circles) and 100% (empty triangles) coverage of branched ligands, and was compared to the total entropy calculate for 80% (circles) and 100% (triangles) coverage of linear ligands. ITC results are presented in black squares.*

As mentioned in the main text, an interplay between the backbone length effect (increasing entropy loss with increasing ligand length) and the branching position effect is observed for methyl branched ligands. Figure S20 presents the binding conformational entropy changes for all available branching positions in alkylthiols with backbone length of 4 to 9 carbons. As the backbone length increases, the entropy loss increases almost regardless the branching positions (odd-even effect is also observed, and is discussed in the next paragraph). Within the set of branched ligands with the same backbone length, the minimal entropy loss is achieved when the branching position is located towards the middle of the chain. While shorter ligands exhibit the highest entropy loss in the iso position, longer ligands demonstrate the highest loss at the 2nd carbon position. As explained in the main text, the steric hindrance induced by methyl branching located at the 2nd carbon position barely affects the end-chain carbons, thereby allowing partial packing of the ligands, which results in higher entropy loss. In addition, the changes in the entropy upon promoting the methyl branching group from the 3rd carbon to the iso- position, are moderated for longer ligands. Taken together, these observations demonstrate the significant role of the backbone length in the overall entropy change.

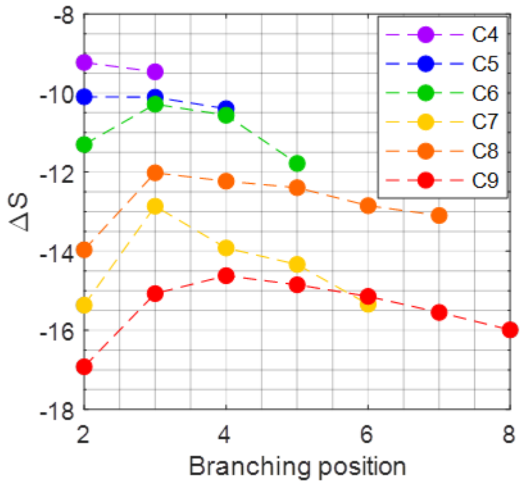

Figure S20. *Conformational entropy calculation for methyl branched alkylthiols. Legend presents the length of the backbone.*

7.2.7. Odd-even effect in branched ligands

As described in the main text, a moderate odd-even effect is observed for 2-methyl branched ligands. Figure S21 presents the conformational entropy and the terminal bond order parameter for linear ligands (4 to 16 carbons) and for branched ligands (4 to 14 carbons) with methyl branching group at the 2nd to 5th position. We chose to expand the length range of investigated ligands in order to better understand the correlation between conformational entropy and the terminal bond order parameter, previously discussed in section 7.2.3.

Generally, we fins that all investigated ligand sets exhibit an odd-even effect. In addition, an inflection point in the entropy is observed. This point is characterized by a moderate change in the entropy followed by an inversion of the odd-even effect. For example, for partial coating of linear ligand, the entropy loss is lower, even for ligands up to a chain with 12 carbons (the inflection point), where similar entropy loss was calculated for chains with 11 to 13 carbons. For longer ligands, the opposite behavior is observed, where even-numbered ligands have higher entropy loss (Figure S21a, pink). The inflection point shifts to longer ligand (14 carbons) for the fully coated system (Figure S21a, yellow). The order parameter of the terminal bond partially reproduces this behavior, where a moderate change in value is observed around the inflection point of the entropy followed by (late) recovery of the odd-even effect (Figure S21b).

Similar behavior is observed for 70% coating of branched ligands, with the inflection point found at the 10-carbon chain (Figure S21c, S20e S20g and S20i, blue). This point shifts to longer chains (12 carbons) for higher coating (Figure S21e, presented for 90% coating of 3-

methyl branched ligand). Here also the order parameter of the terminal bond reproduces the expected odd-even behavior with inflection point at the expected ligand length (Figure S21d, S20f S20h and S20j). This corroborates the previously discussed indication that the odd-even effect in conformational entropy is related to changes in the order parameter of the terminal bond. However, for 4-methyl branched ligands no odd-even effect is observed in the order parameters below the inflection point (Figure S21h). This again suggests that the order parameter is not the sole source for the entropic odd-even behavior, and additional parameters should be considered.

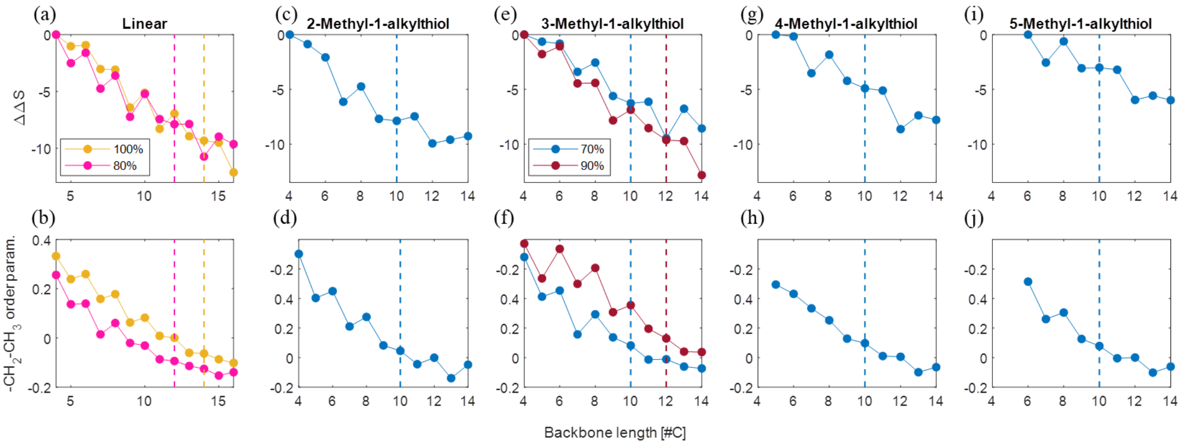

Figure S21. *Odd-even effect in the conformational entropy and the order parameter of the terminal bond for (a-b) full (yellow) and partial (pink) coating of linear ligand, (c-d) 70% coating of 2-methyl-1-alkythiol ligand, (e-f) 70% (blue) and 90% (brown) coating of 3-methyl-1-alkythiol ligand, (g-h) 70% coating of 4-methyl-1-alkythiol ligand, and (i-j) 70% coating of 5-methyl-1-alkythiol ligand. Values of the entropy inflection points are marked with dashed lines with color coding corresponding to the ligand coverage.*

(1) Elimelech, O.; Aviv, O.; Oded, M.; Banin, U. A Tale of Tails: Thermodynamics of CdSe Nanocrystal Surface Ligand Exchange. *Nano Lett.* **2020**, *20* (9), 6396–6403.
(2) Hedenström, E.; Nguyen, B. V.; Silks, L. A. Do Enzymes Recognise Remotely Located Stereocentres? Highly Enantioselective Candida Rugosa Lipase-Catalysed Esterification of the 2- to 8-Methyldecanoic Acids. *Tetrahedron Asymmetry* **2002**, *13* (8), 835–844.
(3) Snow, A. W.; Foos, E. E. Conversion of Alcohols to Thiols via Tosylate Intermediates. *Synthesis (Stuttg).* **2003**, *2003* (4), 509–512.
(4) Anderson, N. C.; Hendricks, M. P.; Choi, J. J.; Owen, J. S. Ligand Exchange and the Stoichiometry of Metal Chalcogenide Nanocrystals: Spectroscopic Observation of Facile Metal-Carboxylate Displacement and Binding. *J. Am. Chem. Soc.* **2013**, *135* (49), 18536–18548.
(5) Wiseman, T.; Williston, S.; Brandts, J. F.; Lin, L. N. Rapid Measurement of Binding Constants and Heats of Binding Using a New Titration Calorimeter. *Anal. Biochem.* **1989**, *179* (1), 131–137.
(6) Fritzinger, B.; Capek, R. K.; Lambert, K.; Martins, C.; Hens, Z. Utilizing Self-Exchange to Address the Binding of Carboxylic Acid Ligands to CdSe Quantum Dots. *J. Am. Chem. Soc.* **2010**, *132*, 10195–10201.
(7) Drijvers, E.; De Roo, J.; Martins, J. C.; Infante, I.; Hens, Z. Ligand Displacement Exposes Binding Site Heterogeneity on CdSe Nanocrystal Surfaces. *Chem. Mater.* **2018**, *30* (3), 1178–1186.
(8) Zhu, C.; Chen, D.; Cao, W.; Lai, R.; Pu, C.; Li, J.; Kong, X.; Peng, X. Facet-Dependent On-Surface Reactions in the Growth of CdSe Nanoplatelets. *Angew. Chemie - Int. Ed.* **2019**, *58* (49), 17764–17770.
(9) George, A.; Choudhary, H. K.; Satpati, B.; Mandal, S. Synthesis, Characterization and Optical Properties of Ligand-Protected Indium Nanoparticles. *Phys. Chem. Chem. Phys.* **2015**, *17* (11), 7109–7113.
(10) Flory, P. J. *Statistical Mechanics of Chain Molecules*; Interscience Publishers: New York, 1969.
(11) Ben-Shaul, A.; Szleifer, I.; Gelbart, W. M. Statistical Thermodynamics of Amphiphile Chains in Micelles. *Proc. Nati. Acad. Sci.* **1984**, *81* (July), 4601–4605.
(12) Szleifer, I.; Ben-Shaul, A. Chain Statistics in Micelles and Bilayers: Effects of Surface Roughness and Internal Energy. *J. Chem. Phys.* **1986**, *85* (9), 5345–5358.
(13) M. A. Carignano; Szleifer, I. On the Structure and Pressure of Tethered Polymer

Layers in Good Solvent. *Macromolecules* **1995**, *28*, 3197–3204.

(14) Feng Tao and Steven L. Bernasek. Understanding Odd−Even Effects in Organic Self-Assembled Monolayers. *Chem. Rev.* **2007**, *107*, 1408−1453.

(15) Wang, Z.; Chen, J.; Oyola-Reynoso, S.; Thuo, M. The Porter-Whitesides Discrepancy: Revisiting Odd-Even Effects in Wetting Properties of n-Alkanethiolate SAMs. *Coatings*. 2015, pp 1034–1055.

(16) Huggins, M. L. Some Properties of Solutions of Long-Chain Compounds. *J. Phys. Chem. C* **1942**, *46* (1), 151–158.

(17) Flory, P. J. Thermodynamics of High Polymer Solutions. *J. c* **1942**, *10*, 51–61.

(18) Rubinstein, M.; Colby, R. H. Thermodynamics of Mixing. In *Polymer Physics*; Oxford University Press: New York, 2003; pp 137–146.

(19) Zhao, G.; Ni, H.; Ren, S.; Fang, G. Correlation between Solubility Parameters and Properties of Alkali Lignin/PVA Composites. *Polymers (Basel).* **2018**, *10* (3).

(20) Kaddour, L. O.; Anasagasti, M. S.; Strazielle, C. Molecular Weight Dependence of Interaction Parameter and Demixing Concentration in Polymer-Polymer-Good Solvent Systems. Comparison with Theory. *Die Makromol. Chemie* **1987**, *188* (9), 2223–2230.

(21) Kamide, K.; Matsuda, S.; Saito, M. Flory Enthalpy Parameter at Infinite Dilution of Polymer Solutions Determined by Various Methods. *Polym. J.* **1988**, *20* (1), 31–43.

(22) Nedoma, A. J.; Robertson, M. L.; Wanakule, N. S.; Balsara, N. P. Measurements of the Composition and Molecular Weight Dependence of the Flory-Huggins Interaction Parameter. *Macromolecules* **2008**, *41* (15), 5773–5779.

(23) Eckelt, J.; Samadi, F.; Wurm, F.; Frey, H.; Wolf, B. A. Branched versus Linear Polyisoprene: Flory-Huggins Interaction Parameters for Their Solutions in Cyclohexane. *Macromol. Chem. Phys.* **2009**, *210* (17), 1433–1439.
